# Interictal spikes during sleep are an early defect in the Tg2576 mouse model of β-amyloid neuropathology

**DOI:** 10.1038/srep20119

**Published:** 2016-01-28

**Authors:** Korey Kam, Áine M. Duffy, Jillian Moretto, John J. LaFrancois, Helen E. Scharfman

**Affiliations:** 1 The Nathan Kline Institute for Psychiatric Research Center for Dementia Research Orangeburg, NY 10962, USA; 2 Department of Physiology and Neuroscience New York University Langone Medical Center New York, NY 10016, USA; 3 Department of Child and Adolescent Psychiatry and Psychiatry New York University Langone Medical Center New York, NY 10016, USA; 4 Graduate Program in Physiology and Neuroscience New York University Langone Medical Center New York, NY 10016, USA.

## Abstract

It has been suggested that neuronal hyperexcitability contributes to Alzheimer’s disease (AD), so we asked how hyperexcitability develops in a common mouse model of β-amyloid neuropathology - Tg2576 mice. Using video-EEG recordings, we found synchronized, large amplitude potentials resembling interictal spikes (IIS) in epilepsy at just 5 weeks of age, long before memory impairments or β-amyloid deposition. Seizures were not detected, but they did occur later in life, suggesting that IIS are possibly the earliest stage of hyperexcitability. Interestingly, IIS primarily occurred during rapid-eye movement (REM) sleep, which is notable because REM is associated with increased cholinergic tone and cholinergic impairments are implicated in AD. Although previous studies suggest that cholinergic antagonists would worsen pathophysiology, the muscarinic antagonist atropine reduced IIS frequency. In addition, we found IIS occurred in APP51 mice which overexpress wild type (WT)-APP, although not as uniformly or as early in life as Tg2576 mice. Taken together with results from prior studies, the data suggest that surprising and multiple mechanisms contribute to hyperexcitability. The data also suggest that IIS may be a biomarker for early detection of AD.

Alzheimer’s disease (AD) is a devastating and progressive disease where few treatments are successful^1^. One possible contribution to AD that is often not considered as a target for treatment is epileptiform discharges and seizures, which are particularly common in familial forms of AD^2^,^3^,^4^. However, antiseizure drugs (ASDs) have been reported to improve memory impairment in patients^5^, as well as memory deficits in mouse models of β-amyloid neuropathology^6^. Therefore a better understanding of hyperexcitability in AD is potentially important^7^.

In this study, we investigated a widely used mouse model, Tg2576 mice, where mice overexpress human amyloid precursor protein (APP) with the mutation found in a familial form of AD in Sweden (APPSwe)^8^. The Swedish mutation greatly facilitates the β-site cleavage of APP, leading to increased levels of the toxic metabolite amyloid-β (Aβ)^9^. In contrast to Tg2576 mice, most mouse models used to study hyperexcitability in AD contain more than 1 mutation in either APP cleavage sites or the secretases that cleave APP (e.g. APPSwe/Indiana, APPSwe/presenilin 1 (PS1) Δe9), resulting in accelerated β-amyloid deposition^10^.

Using the Tg2576 mouse at a very young age (5 week-old), long before β-amyloid deposition^8^,^11^,^12^, we found a characteristic hallmark of epilepsy - interictal spikes (IIS). IIS reflect sudden, large, synchronous depolarizations in principal cells that last only a fraction of a second^13^. A recent publication found brief IIS occur in young (6 week-old) Tg2576 mice as well^14^. Interestingly, we found that IIS in 5 week-old Tg2576 mice occurred primarily during the rapid eye movement (REM) stage of sleep. Epileptiform discharges and seizures were absent at 5–7 weeks of age, but did occur at older ages. The results suggest that IIS during sleep are a very early stage of hyperexcitability in Tg2576 mice.

We clarify mechanisms contributing to IIS, by demonstrating that a muscarinic cholinergic receptor antagonist reduces IIS, which is surprising in light of the literature suggesting cholinergic function gradually deteriorates in AD. We also show that nicotinic antagonists do not have this effect, and the antiseizure drug levetiracetam reduces IIS. Furthermore, mice with overexpression of WT-APP have some IIS or IIS-like events in their EEG, although not as uniformly or as young as 5 week-oldTg2576 mice. We suggest that multiple mechanisms contribute to IIS and that IIS could be useful as an early biomarker.

## Results

### Interictal-like spikes are present in 5 week-old Tg2576 mice but not age-matched WT littermates

**Figure 1:**
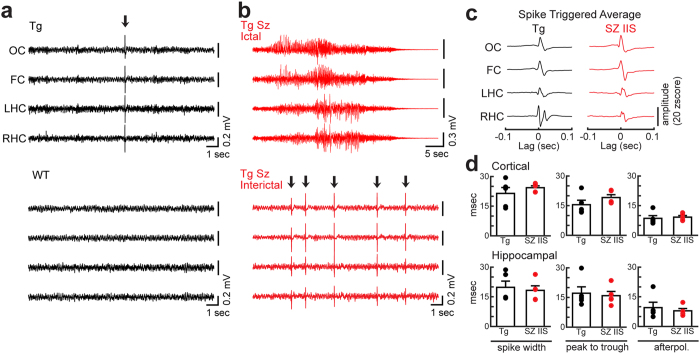
IIS occur in 5 week-old Tg2576 mice but not WT littermates. (**a**) Representative EEG traces during REM sleep from a 5 week-old Tg2576 (Top, TG) and WT mouse (Bottom, WT). Arrow indicates an IIS in the Tg2576 mouse. OC = occipital cortex; FC = frontal cortex; LHC = left hippocampus; RHC = right hippocampus. (**b**) A representative EEG recording of a seizure (Top, TG SZ Ictal) in a 7 month-old Tg2576 mouse. The subsequent interictal period from the same mouse (Bottom, TG SZ Interictal). Arrows indicate IIS. (**c**) Spike-triggered average (STA) waveforms from a 24 hour recording period of the 5 week-old Tg2576 mouse without seizures (TG, same mouse as **a**) and a Tg2576 mouse with a seizure (SZ IIS, same mouse as **b**). STA waveforms were similar whether seizures were detected or not. (**d**) Characteristics of IIS for 5 week-old Tg2576 mice (TG, n = 5 mice) and older Tg2576 mice with a spontaneous seizure (SZ IIS, n = 5 mice). There was no effect of group (5 week-old mice without seizures vs. older mice with seizures) in cortical IIS (Top; two-way ANOVA, F(1,26)1.444, p = 0.240) or hippocampal IIS (Bottom; two-way ANOVA, F(1,26)0.482 p = 0.494).

We performed simultaneous video-EEG using cortical (left frontal and right occipital cortex) and bilateral dorsal hippocampal electrodes in freely behaving Tg2576 and WT littermates. We began recording at 5 weeks of age, prior to the earliest behavioral deficits that have been reported (at 3–6 months^12^,^15^,^16^) or the earliest age when β-amyloid deposition occurs (at approximately 6 months in Tg2576 mice^11^). To assess all behavioral states (e.g. spontaneous exploration, sleep), we recorded for 24 hours continuously in the home cages. Remarkably, we found large amplitude, transient spike-like events occurring synchronously in all cortical and hippocampal leads and specifically during sleep (Fig. 1a). These spikes were present in all transgenic mice (n = 9) and absent in all WT littermates (n = 17; Fig. 1a, Supplementary Table 1). IIS frequency was consistent from day to day, based on continuous recordings for 5 days, starting at 5 weeks of age (Supplementary Fig. 1a). These data suggest that Tg2576 mice have abnormal excitability, reflected by IIS, long before β-amyloid deposition.

The term IIS is used here to refer to the large transient spikes because they resembled the IIS that occurred between spontaneous seizures (i.e. between ictal events; Fig. 1b–d; Supplementary Fig. 1b–d) which occurred later in life. IIS in Tg2576 mice also resembled IIS between seizures in a previous study of mice injected with the convulsant kainic acid, recorded with the same procedures^17^. Notably, at 5 weeks of age, no seizures were detected in Tg2576 mice, either in the 5 week-old mice recorded for 24 hours (n = 9), or 5 week-old mice recorded continuously for 2 weeks (from 5 to 7 weeks of age; n = 6 mice).

### IIS primarily occur during REM sleep in 5 week-old Tg2576 mice

**Figure 2:**
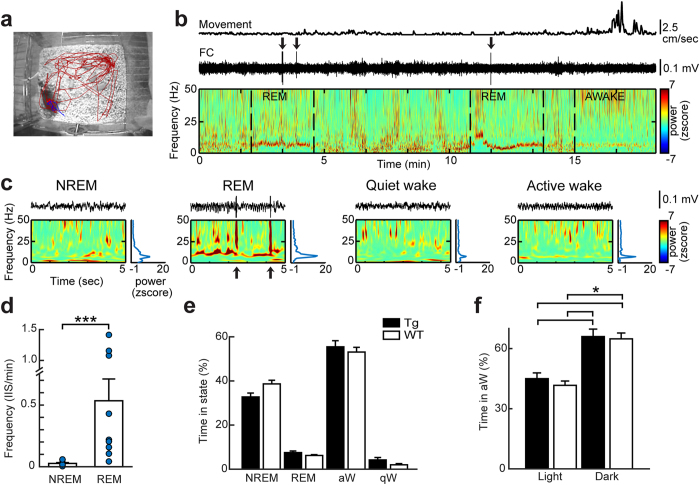
IIS in 5 week-old Tg2576 mice occur predominantly within REM sleep. (**a**) An example of positional tracking used to monitor mouse movement in the home cage. The red line marks animal position in the previous 10 minutes while the blue line marks position in the last 60 seconds. (**b**) A 20 minute-long EEG recording from a 5 week-old Tg2576 mouse shows detected movement (Top), EEG (Center, frontal cortical (FC) electrode) and spectrogram (Bottom). Note that IIS (arrows) occur in the behavioral states without movement and power is high in the theta frequency band, associated with REM sleep (for definition of REM sleep and other behavioral states, see Methods). Dotted lines demarcate state transitions. (**c**) Representative 5 second-long EEG traces from **b** illustrate each behavioral state. For each behavioral state (from left to right, NREM, REM, quiet wakefulness, active wakefulness) the FC recording of the EEG is shown at the top, with the spectrogram corresponding to it shown below. To the right is the power spectrum. Note that IIS (arrows) occur in REM sleep. (**d**) Mean IIS frequency per minute for 24 hour-long recordings of 5 week-old Tg2576 mice. Mean IIS frequency in REM sleep epochs was greater than mean IIS frequency in NREM sleep epochs (n = 9; Mann-Whitney test, U = 47, p = 0.0002). WT mice did not exhibit IIS (n = 9 mice, Supplementary Table 1). In this figure and all others, *p < 0.05; **p < 0.01; ***p < 0.001. (**e**) There was no effect of genotype on the time spent in each behavioral state per 24 hour-long recording for 5 week-old Tg2576 (n = 9) and age-matched WT mice (n = 5; two-way ANOVA, F(1,55) < 0.001, p = 0.992). (**f**) For 5 week-old Tg2576 (n = 9) and WT mice (n = 5), there was no effect of genotype on the time spent in active wakefulness during the light or dark periods (two-way ANOVA, F(1,27)0.371, p = 0.548). However, there was a significant effect of light or dark period, with all animals spending more time in active wakefulness during the dark period (two-way ANOVA, F(1,27)36.262, p < 0.001; post-hoc test, p < 0.05).

IIS were observed predominantly within REM sleep. To quantify this observation, we defined behavioral state for the entire 24 hour-long recording at 5 weeks of age for all mice. Behavioral state was defined as active wakefulness (e.g., spontaneous exploration), quiet wakefulness (transient behavioral arrest), non-REM (NREM) sleep or REM sleep (Fig. 2a–c). The classification of each behavioral state was based on movement and spectral features of the EEG (see Methods and Supplementary Figs. 2 and 3). The results showed that IIS in 5 week-old Tg2576 mice occurred primarily in REM sleep (Fig. 2d, Supplementary Fig. 4a,b). IIS were completely absent from wakeful behavior (Fig. 2b–d, Supplementary Fig. 4a,b). Importantly, there were no significant differences between Tg2576 and WT mice in the time spent in each behavioral state during a 24 hour-long period at 5 weeks of age (Fig. 2e). There also were no genotypic differences in the behavioral states that occurred during the light vs. dark part of the 24 hour-long recording (Fig. 2f, Supplementary Fig. 4c). These findings are consistent with the observations that circadian impairments in Tg2576 mice occur only at older ages (12–15 months of age but not at younger ages 2–5 months^18^,^19^). They identify a striking abnormality of neural activity during sleep in young Tg2576 mice.

Although the Tg2576 mouse has elevated Aβ levels at young ages (2 months-old^16^), a role of human WT-APP overexpression in generation of IIS is also important to consider because it has been suggested to play a role in hyperexcitability in the APPSwe/Ind mouse^20^. To determine the potential role of full-length human APP on the generation of IIS, we performed 24 hour-long video-EEG recordings in APP51 mice, which overexpress human WT-APP, devoid of familial AD mutations^21^ (see Methods). IIS were present in older mice but not at 5 weeks of age (Supplementary Table 1, Supplementary Fig. 5), in contrast to Tg2576 mice. Therefore, WT-APP does not appear to contribute to IIS at 5 weeks of age.

Notably, IIS or IIS-like events (IIS recorded on 3 channels instead of 4; Supplemental Fig. 5) were present in all of the APP51 mice at 7 months of age. Remarkably, the IIS were similar to Tg2576 mice because they were present only in REM sleep. All IIS were REM specific - those recorded on all 4 channels or only 3 channels. The results suggest that full-length WT-APP could contribute to IIS at 7 months of age.

### IIS increase with age and emerge in additional behavioral states besides REM sleep

**Figure 3:**
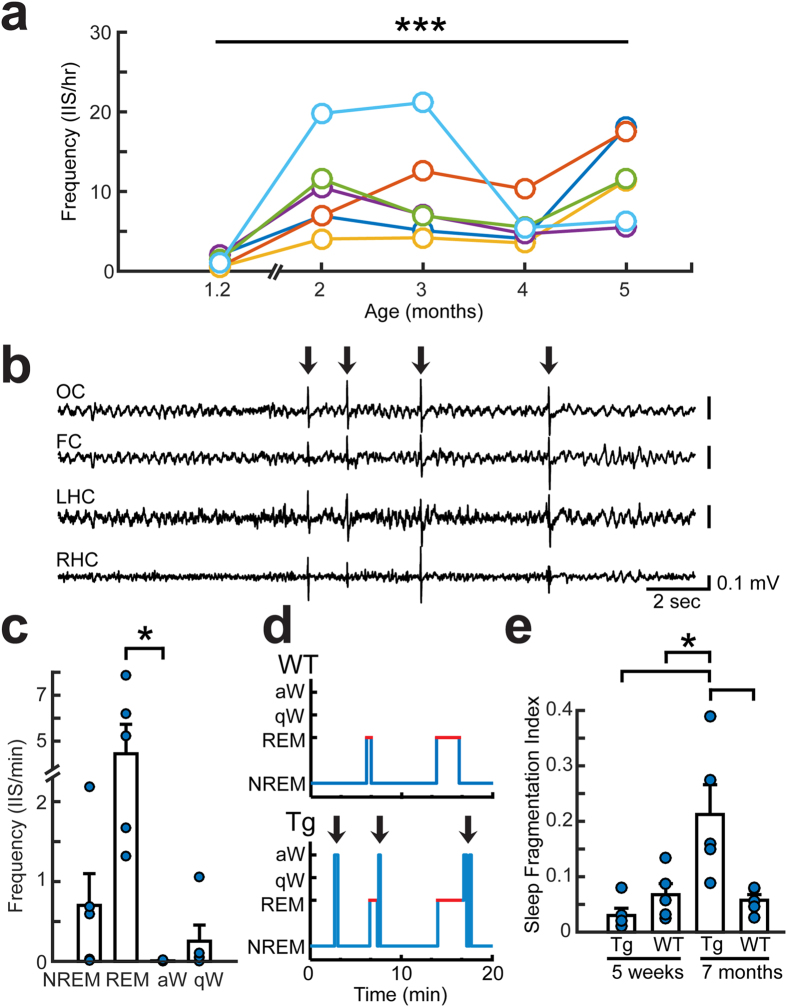
IIS increase with age in Tg2576 mice. (**a**) Mean IIS frequency for 24 hour-long recordings that were made at 1.2, 2, 3, 4, and 5 months of age. Each mouse is denoted by a different color. There was a significant effect of age (repeated-measures ANOVA, F(4,35)8.514, p < 0.001). (**b**) A representative EEG recording during REM sleep in a 7 month-old Tg2576 mouse. Arrows mark IIS. (**c**) IIS frequency in each behavioral state at 7 months of age (n = 5). There were significant differences in IIS frequency between different behavioral states (Kruskal-Wallis test, H(4,19)24, p = 0.009). IIS frequency in NREM and REM were not significantly different at 7 months of age, unlike 5 weeks of age (see Fig. 2). At 7 months of age, IIS frequency in REM was only significantly different from IIS frequency in active wakefulness (post-hoc test, p < 0.05). (**d**) An example of a hypnogram comparing sleep episodes in a Tg2576 and WT mouse at 7 months of age. Arrows mark arousals from sleep, which were more frequent in the Tg2576 mouse at 7 months-old compared to age-matched WT mice. (**e**) The sleep fragmentation index (SFI) in 5 week-old (n = 5) and 7 month-old mice (n = 5). There was a significant increase in SFI in Tg2576 mice with age (two-way ANOVA, F(1,19)8.379, p = 0.018, post-hoc test, p < 0.05). In addition, 7 month-old Tg2576 mice had a greater SFI than 5 week-old and 7 month-old WT mice (post-hoc test, p < 0.05). There was an interaction between age and genotype (F(1,19)10.436, p = 0.005) but no effect of genotype alone (F(1,19)3.880, p = 0.066).

Given the progressive β-amyloid neuropathology in Tg2576 mice, we next asked if IIS showed an age-dependent exacerbation. To address this issue, we performed video-EEG recordings for 24 hours every 3–4 weeks from 5 weeks to 5 months of age. We found an age-dependent increase in IIS frequency during the initial 2–3 months, followed by variability in IIS frequency (Fig. 3a,b). WT littermates did not have IIS at any age (tested from 5 weeks to 23 months; Supplementary Table 1).

As animals aged, IIS continued to occur in sleep but also emerged in quiet wakefulness (Fig. 3c). This change was unrelated to increased duration of time spent in quiet wakefulness because the fraction of time spent in each behavioral state remained stable with age (compare Fig. 2f and Supplementary Fig. 6). However, there were more frequent arousals from sleep in 7 month-old Tg2576 mice compared to age-matched WT littermates (Fig. 3d,e), reflected by the sleep fragmentation index (SFI). Consistent with this finding, fragmented sleep has been observed in Tg2576 mice previously at 12 months but not 2 months of age^18^. Thus, the emergence of IIS in behavioral states other than sleep coincided with frequent arousals from sleep and therefore may be related to circadian or sleep-associated abnormalities that develop with age in the Tg2576 mice.

Similar to 3–5 month-old Tg2576 mice (Fig. 3a), older Tg2576 mice (12–23 months) also varied in their IIS frequency. These older mice were recorded for 24 hour-long recording sessions. In mice 12–23 months old, IIS frequency was highly variable (16.3 ± 24.1 IIS/hr, n = 3). To determine spontaneous seizure frequency in Tg2576 mice at these older ages, we performed 2 week-long continuous video-EEG recordings. In 5 mice that were 8–24 months of age, we found 1 seizure during the 2 week-long recording in 2 mice and no seizures in the other 3 mice. Because IIS did not gradually build up in the minutes or hours before or after the seizure (data not shown), the possibility that IIS frequency changes because of seizures seems unlikely.

Together these data suggest that IIS become more frequent until approximately 3 months of age. After that age, IIS become variable in frequency and emerge in quiet wakefulness. This variability in both frequency and state-dependence may be a reflection of the increasing levels of Aβ and the many functional and structural deficits that have been reported after 3 months of age in Tg2576 mice^15^,^16^.

### A contribution of the cholinergic system to IIS of Tg2576 mice

#### Theta rhythm

**Figure 4:**
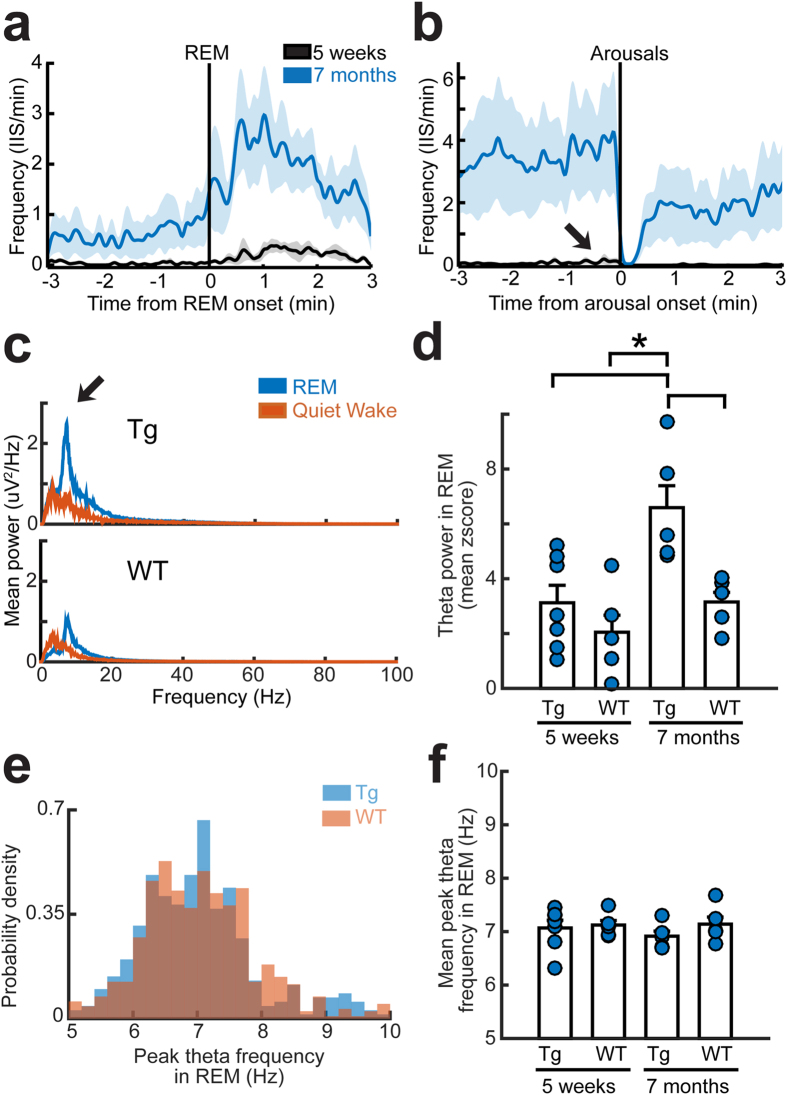
Characteristics of IIS in REM sleep suggest a contribution of the cholinergic system. (**a**) IIS frequency is shown as average perievent time histograms (PETHs) in the 3 minutes before and after REM onset. Data from 5 week-old (black; n = 6) and 7 month-old mice (blue; n = 5) are shown, with the means as solid lines and S.E.M. as shaded colors (5 week-old mice, gray; 7 month-old mice, light blue). IIS increased at the start of REM sleep at both ages. (**b**) The average PETHs for the 3 minutes before and after arousal from sleep in Tg2576 mice at 5 weeks of age and 7 months of age. Same animals and color legend as **a**. IIS frequency decreased during arousals. (**c**) Top: Mean power spectra of all REM sleep epochs (blue) or quiet wakefulness epochs (red) in a 24 hour period in a representative 7 month-old Tg2576 (top) and 7 month-old WT mouse (bottom). Note greater power in the frequencies corresponding to theta in the Tg2576 mouse (arrow). (**d**) Quantification of theta power for all REM sleep epochs in a 24 hour-long recording in 5 week-old WT (n = 5) and Tg2576 mice (n = 7), and 7 month-old Tg2576 (n = 5) and WT mice (n = 5). There was an effect of age (two-way ANOVA, F(1,21)10.253, p = 0.0049) and genotype (F(1,21)9.998, p = 0.0054) with 7 month-old Tg2576 mice having greater theta power compared to all other groups (post-hoc tests, p < 0.05). (**e**) Histograms show the distribution of peak theta frequencies in all REM sleep epochs during a 24 hour recording session in a representative Tg2576 and WT mouse at 7 months of age. Peak frequencies were similar (quantified in **f**). (**f**) There were no significant effects of age or genotype on peak theta frequency during REM sleep (same mice as **d**; two-way ANOVA, age effect: F(1,21)0.234, p = 0.630; genotype effect: F(1,21)1.044, p = 0.320).

We next considered a role of the cholinergic system in IIS, an idea that arose from two characteristics of the cholinergic system. First, REM sleep is associated with an increase in the activity of cholinergic projections to the hippocampus and cortex^22^. Second, muscarinic cholinergic agonists^23^ as well as acetylcholine (ACh)^24^ can cause hyperexcitability and seizures in rodents. Although the long-standing cholinergic hypothesis for AD suggests that there is a decay in cholinergic pathways in AD^25^, there could be high cholinergic tone before the decay. In support of this hypothesis, IIS frequency increased with the onset of REM sleep (Fig. 3b), determined by perievent time histograms (PETHs; Fig. 4a). The increase in IIS frequency at the onset of REM sleep occurred in the 30–60 seconds after the start of REM (Fig. 4a, Supplementary Fig. 7a), which is important because ACh levels normally rise in the brain at this time^26^,^27^,^28^. The PETHs were most striking at 7 months of age because IIS frequency was low at earlier ages (Fig. 4a, Supplementary Fig. 7a).

In contrast to the onset of REM sleep, IIS decreased during REM to NREM sleep transitions, when ACh levels fall^26^ (Supplementary Fig. 7b). In addition, IIS frequency decreased dramatically during arousal from sleep (i.e. the transition from sleep to wakefulness; Fig. 4b, Supplementary Fig. 7c).

In light of the contribution of the cholinergic system to hippocampal theta rhythm^29^, we computed theta power and peak theta frequency. Theta power was generally higher in Tg2576 mice but theta frequency was not. Thus, Tg2576 mice had greater theta power than WT mice at 7 months of age (Fig. 4c–f). In 5 week-old mice the mean value was larger for Tg2576 mice than WT, but statistical comparisons showed that the p value was not significant (Fig. 4c,d). When hippocampal recordings were analyzed instead of cortical EEG, the greater theta power in 5 week-old Tg2576 compared to WT mice was closer to significance (p = 0.054) but did not strictly meet criterion (p < 0.05). There were no detectable group differences in peak theta frequency at either age (Fig. 4e,f), suggesting changes in power rather than a major disruption in theta rhythm. Consistent with specific changes in theta power rather than frequency, frequency bands other than theta did not show differences between Tg2576 and WT mice (Fig. 4c). Furthermore, theta power in NREM sleep was not different between Tg2576 and WT mice (Supplementary Fig. 8). There also were no differences in NREM delta power, a defining feature of NREM sleep [two-way ANOVA, effect of genotype: F(1,21)1.965, p = 0.178; effect of 5 week-old vs 7 month-old age: F(1,21)0.255, p = 0.620)].

### Choline acetyltransferase (ChAT) and c-fos immunoreactivity (ir)

**Figure 5:**
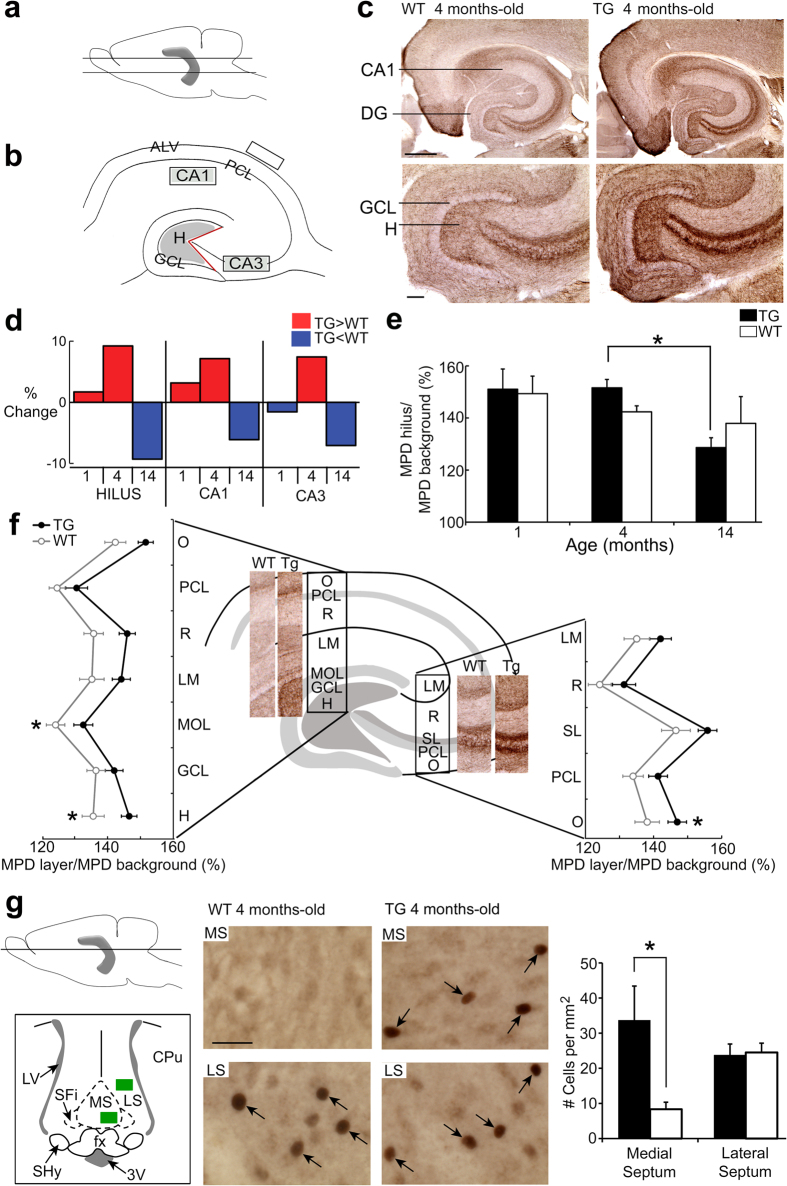
Increased ChAT and c-fos immunoreactivity (ir) in Tg2576 mice. (**a**) Hippocampal (gray) schematic with lines indicating where sections were chosen. (**b**) Schematic showing where mean pixel density (MPD) was measured (gray; hilus; CA1b stratum radiatum; CA3b stratum lucidum-oriens). White matter was used to measure background (rectangle). ALV = alveus; PCL = pyramidal cell layer; GCL = granule cell layer; H = hilus. (**c**) Representative ChAT-ir in 4 month-old WT and Tg2576 mice (TG). Top: Calibration = 500 μm. Bottom: Calibration = 100 μm. DG = dentate gyrus. (**d**) Comparisons of %MPD ([MPD region/MPD background]x100) for hilus, CA1, and CA3 at 1, 4, and 14 months. Then %MPD for WT was subtracted from %MPD for Tg2576 mice. Tg2576 mice had greater ChAT-ir than WT (red) at 4 months but less (blue) at 14 months. (**e**) Hilar MPD quantification. There was a region (three-way ANOVA, F(1,114)70.951; p < 0.0001) and age effect (F(1,114)14.533; p < 0.0001), with region x age (F(1,114)5.720; p = 0.0004) and genotype x age (F(1,114)4.362; p = 0.015) interactions. There was an age effect (two-way ANOVA, F(1,38)5.195; p = 0.101). One-way ANOVA showed an age effect for Tg2576 mice (F(2,22)7.948; p = 0.0025), with 4 months-old hilar ChAT-ir greater than 14 months-old (post-hoc test, p < 0.05). There was no WT age effect (ANOVA, F(2,16)0.920; p = 0.419). (**f**) ChAT-ir laminar analysis of CA1/DG (left), and CA3 (right) for 4 month-old WT (gray) and Tg2576 mice (black). There was a genotype effect (two-way ANOVA, F(1,24)6.373, p < 0.0186), with greater ChAT in the molecular layer (MOL), hilus and stratum oriens (O) of CA3 in Tg2576 mice (post hoc tests, p < 0.05). LM = stratum lacunosum-moleculare; R = stratum radiatum. (**g**) Left: Top. Schematic illustrating where sections were chosen from medial septum (MS) and lateral septum (LS). Bottom. Green rectangles show where in the MS and LS c-fos-ir cells were counted. 3 V = third ventricle, CPu = caudate putamen, fx = fornix, LV = lateral ventricle, SFI = septofimbrial nucleus, SHy = septo-hypothalamic nucleus. Center: Representative c-fos-ir in the MS and LS of 4 month-old WT and Tg2576 mice. Calibration = 30 μm. Right: Bar-graph showing c-fos-ir density in the MS and LS of Tg2576 (black; n = 6) and WT (white; n = 6) mice. Two-way ANOVA showed a genotype effect (F(1,20)5.139, p = 0.0346) and a genotype x region interaction (F(1,20)5.814, p = 0.0256), with greater c-fos-ir cells in the Tg2576 MS (post-hoc tests, p < 0.05).

To further analyze cholinergic changes in Tg2576 mice, an antibody was used to choline acetyltransferase (ChAT), the rate-limiting enzyme for ACh synthesis in neurons. ChAT-immunoreactivity (ir) was high in Tg2576 mice at 1 and 4 months of age compared to 14 months of age, which was particularly clear in the hippocampus, possibly due to the laminar organization of the primary cholinergic input, the septohippocampal projection (Fig. 5a–f). Based on mean pixel density, a detailed laminar analysis showed that 4 month-old Tg2576 mice exhibited greater ChAT-ir than WT mice in the layers containing septohippocampal terminals (Fig. 5f). Mean pixel density measurements of selected regions also suggested that ChAT-ir significantly declined at 14 months of age in Tg2576 mice but not WT mice (Fig. 5d,e). The decline in Tg2576 ChAT-ir with age is consistent with prior reports of abnormal/deteriorating cholinergic fibers in Tg2576 mice at ages older than 1 year^18^,^30^. As a caveat, it is important to note the degree that mouse cholinergic neurons replicate changes in human cholinergic neurons in AD has been criticized^31^.

Interestingly, genotypes were similar in the dorsal striatum, where cholinergic neurons are intrinsic. Thus, in dorsal striatum, there were no genotypic differences in the number of cholinergic neurons or mean pixel density (Supplementary Fig. 9a,b) in the same tissue where there was greater ChAT-ir in hippocampus of Tg2576 mice (Fig. 5). However, Tg2576 mice exhibited greater ChAT-ir in some parts of the cortex such as retrosplenial cortex (RSC) which has been implicated in AD^32^,^33^. In RSC, Tg2576 mice exhibited greater ChAT-ir than WT in the superficial layers (Supplementary Fig. 9c), the layers targeted by the medial septum (MS)^34^ and the site where Aβ deposition occurs in the 3xTg mouse model of AD^35^.

Because the MS makes a major contribution to hippocampal, cortical and RSC ChAT-ir^31^, we next addressed ChAT-ir in MS in the same tissue sections where the projections showed elevated ChAT-ir. The results showed that there was no detectable cell loss or unusual ChAT-ir in the MS of 4 month-old Tg2576 mice (Supplementary Fig. 9a,b). We next considered the hypothesis that MS neurons in 4 month-old Tg2576 mice were highly active compared to WT, possibly during sleep. To test this hypothesis, 4 month Tg2576 and WT mice were compared after they had been sleeping for >2 hrs. An antibody to c-fos was used as a marker of neuronal activity. The results showed that young Tg2576 mice had greater numbers of c-fos-ir nuclei in the MS compared to WT mice (Fig. 5g). Yet areas outside the septum were not different, even those closeby such as the lateral septum (Fig. 5g). Taken together, the upregulation of ChAT-ir in 4 month-old Tg2576 mice, primarily in cholinergic axons that project to hippocampus and cortex, and the elevated c-fos-ir in MS of 4 month-old Tg2576 mice, suggest abnormalities in the septohippocampal neurons that could be due to overactivity of MS neurons.

### IIS are reduced by muscarinic receptor antagonism

Considering the data suggesting that MS neurons were not deteriorating, but could be more active in young Tg2576 mice than WT mice, we determined whether antagonists of cholinergic receptors would reduce IIS frequency. We also examined the effect of donepezil, a cholinesterase inhibitor that is used to treat AD. Levetiracetam was also tested, because it is an anti-seizure medication that has been previously shown to reduce epileptiform activity in a mouse model of β-amyloid neuropathology (APPSwe/Indiana; “J20” mice)^6^ and improved cognition in patients with mild cognitive impairment^5^.

**Figure 6:**
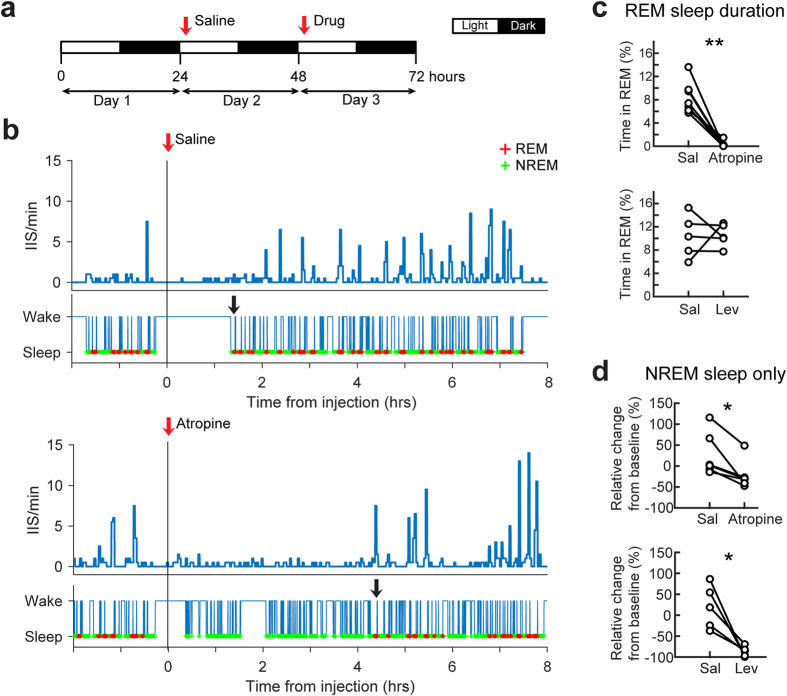
Pharmacology of IIS in Tg2576 mice. (**a**) Experimental timeline. Animals were recorded for 3 days. Vehicle (saline) was injected 1 hour after the start of the light period (white bar) on Day 2 (red arrow). On Day 3, drug was injected 1 hour after the start of the light period (red arrow). (**b**) Top: An example of the responses to saline injection. IIS frequency changes after saline injection (red arrow) are shown as a histogram (2 minutes per bin). Behavioral state (sleep or wake) is shown below the histogram. Red (+) symbols indicate REM sleep and green (+) symbols represent NREM sleep. Bottom: A representative response to injection of the cholinergic muscarinic receptor antagonist atropine (red arrow). Both saline and atropine injection rapidly reduced IIS frequency because of the interruption of sleep induced by the injection. However, IIS resumed when REM sleep returned (black arrows). (**c**) The effects of injection on the percent of time spent in REM sleep is shown for all animals. Atropine reduced REM sleep more than saline in the 0–4 hours after drug injection (paired t-test, n = 7, p = 0.002) but there was no consistent effect of levetiracetam on REM sleep (paired t-test, n = 5, p = 0.929). Because atropine decreased REM sleep, effects of atropine were analyzed in NREM sleep in part. (**d**) Atropine significantly reduced IIS frequency in NREM sleep compared to saline (paired t-test, n = 7, p = 0.015). Levetiracetam also suppressed IIS frequency compared to saline (paired t-test, n = 5, p = 0.017). The effects of drugs on IIS frequency were defined as the mean IIS frequency in the 0–4 hours after injection compared to the mean baseline IIS frequency. Mean baseline IIS frequency was calculated from all NREM sleep epochs during the 24 hour period defined as Day 1 (see **a**).

We used a 3 day-long experimental design with continuous video-EEG (Fig. 6a). After the baseline recording period (24 hours-long; Day 1), saline (vehicle) was injected at the start of the light period (start of Day 2, Fig. 6a), a time that would provide the greatest chance to capture natural sleep (Supplementary Fig. 6c,d). The injection of the drug occurred 24 hours-later, at the start of Day 3 (Fig. 6a). This experimental design allowed us to evaluate baseline activity, the response to vehicle, and the response to drug within the same animal.

The muscarinic antagonist atropine suppressed IIS in the first 4 hours after administration compared to the first 4 hours after saline injection (Fig. 6b; Supplementary Fig. 10a). However, REM sleep was absent in the 4 hours after atropine injection (Fig. 6b,c), which has been shown previously^36^,^37^ and is consistent with the role of the cholinergic system in REM sleep. Therefore, the effect of atropine was further analyzed in NREM sleep. Seven to 12 month-old mice were used because IIS frequencies in NREM sleep were robust at these ages. Atropine reduced IIS frequency in NREM sleep, supporting the hypothesis that IIS were dependent on muscarinic cholinergic receptors (Fig. 6d). Levetiracetam also reduced IIS frequency in NREM sleep (Fig. 6d, Supplementary Fig. 10b), consistent with prior findings in other mouse models of β-amyloid neuropathology mentioned above.

The nicotinic antagonist mecamylamine induced a slight but significant increase in IIS frequency during the first 4 hours after drug injection (Supplementary Fig. 10c). In contrast, there was no detectable effect of the α7 receptor antagonist methyllycaconitine (Supplementary Fig. 10c). The results suggest that there could be an alteration in nicotinic receptor function in the Tg2576 mice, which indeed has been reported^30^, but the greatest effect on IIS appeared to be muscarinic receptor antagonism.

Interestingly, the cholinesterase inhibitor donepezil did not significantly affect IIS (Supplementary Fig. 10d). These data are consistent with the hypothesis that cholinergic tone was high in the young Tg2576, so increasing ACh levels by administering donepezil had no detectable effect.

Throughout the pharmacological experiments we noticed that some Tg2576 mice had particularly high IIS frequencies before drug administration. These animals appeared to be the mice that had the greatest reduction in IIS by atropine. To quantify this relationship, we asked if there was a positive correlation between the effect of drug and the IIS frequency prior to drug administration. Indeed, there was a significant positive correlation (Supplementary Fig. 10e). In contrast, the correlation was not significant for levetiracetam (Supplementary Fig. 10f). These findings suggest that animals with the highest IIS frequencies had the greatest reduction in IIS frequency by atropine, whereas levetiracetam reduced IIS in all animals. Because of these differences between atropine and levetiracetam, IIS may have non-cholinergic mechanisms that explain the additional effects of levetiracetam. Indeed, non-cholinergic mechanisms have been proposed for different mouse models of β-amyloid neuropathology, including reduced expression of sodium channels in parvalbumin-expressing GABAergic neurons^38^, or a role of human WT-APP and its metabolites^20^,^39^,^40^.

## Discussion

**Figure 7:**
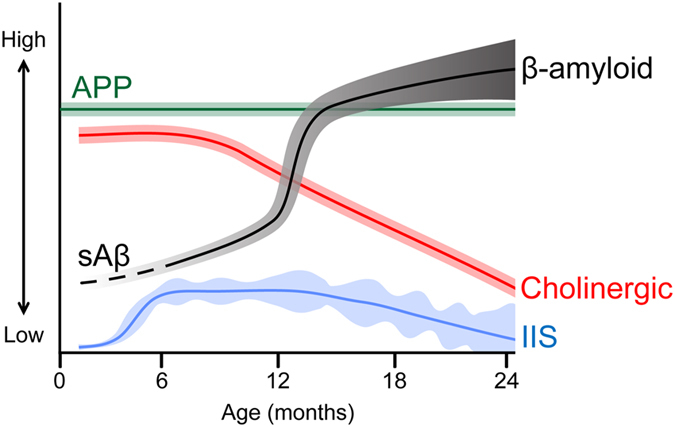
A model of the progressive changes in the Tg2576 mouse with age. (**a**) A schematic summarizes the hypothesis that Tg2576 mice have increased cholinergic tone very early in life with concurrent neuronal hyperexcitability, resulting in IIS during sleep. Blue: Mean IIS frequency (line) and variance (shading) increases with age and becomes variable. Red: Cholinergic markers (e.g. ChAT-ir) are elevated early in life and then decline^18^,^30^. Gray: Aβ levels are indicated by the line, which increase throughout life. Initially the line is dotted and light gray, reflecting the dominance of soluble Aβ (sAβ). The line becomes solid and dark gray as β-amyloid deposition increases.

By investigating neuronal hyperexcitability in young Tg2576 mice prior to β-amyloid deposition, we found that IIS occur at just 5 weeks of age, confirming a prior study^14^. Remarkably, we found that IIS occur primarily during REM sleep. As animals reached 2–3 months-old, IIS increased in frequency within REM sleep, and in subsequent months IIS also increased in other behavioral states. At 7 months of age, IIS increased in frequency at REM onset, theta power was significantly higher during REM relative to 5 weeks of age, suggesting high cholinergic tone rather than low cholinergic tone. Consistent with those findings, ChAT-ir was higher at 1–4 months compared to older ages. Reduction of IIS by the cholinergic antagonist atropine and a drug that reduces excitability, levetiracetam, supported that hypothesis. Interestingly, nicotinic receptor antagonists either did not reduce IIS or led to a small increase in IIS, suggesting that muscarinic receptors (or an abnormal balance of muscarinic/nicotinic receptor function) have the greater influence on IIS. The lack of effect of the cholinesterase inhibitor donepezil supports the hypothesis that cholinergic tone was already high in young Tg2576 mice, so that increasing it further had little effect. The results shown here suggest a refinement of the cholinergic hypothesis of AD: instead of gradual deterioration there may be a phase of high cholinergic tone that precedes the decline (Fig. 7).

### IIS are an early abnormality

Our data show that large amplitude fast transients similar to IIS in animals with epilepsy occurred early in life in Tg2576 mice, and corroborate a recent study^14^. This is the earliest sign of *in vivo* hyperexcitability that has been identified in these mice. However, there are many abnormalities that have been reported in Tg2576 mice, although most studies do not examine mice at 5 weeks of age. At 2 months of age, there is a decline in spines of the principal cells of the dentate gyrus and decreased long-term potentiation (LTP) of the major afferent pathway^12^. In a study we conducted, several structural and functional abnormalities were present in the entorhinal cortex (EC) at 2–4 months of age and one of them was repetitive field potentials in the EC of combined hippocampal-EC slices^16^, supporting the hypothesis of hyperexcitability at early ages in the Tg2576 mouse. However, only abnormal evoked responses were observed, not spontaneous phenomenon like IIS. The absence of spontaneous IIS-like events in slices is notable because they can be recorded in hippocampal slices in response to GABA_A_ receptor antagonists or convulsants^41^, suggesting that the IIS that we recorded *in vivo* in the current study are not reproduced in slices. Instead, IIS may require behavioral states (e.g. sleep) or projections that span long distances (e.g. cholinergic).

Other early changes that have been reported in young Tg2576 mice include impaired olfaction at 3–4 months of age^15^ and impairments in the object placement task at 3–4 months of age^16^. Additional studies have shown that there is altered olfactory connectivity in Tg2576 mice as early as postnatal day 10^42^. Therefore, IIS may be the first sign of hyperexcitability, but other early abnormalities in Tg2576 mice are present.

Considering the evidence that β-amyloid deposition begins at approximately 6 months of age in Tg2576 mice^11^, our results support the view that hyperexcitability precedes β-amyloid deposition^40^,^43^,^44^,^45^,^46^. However, we cannot conclude that hyperexcitability precedes elevation of all forms of Aβ, because we previously found that Aβ40 and Aβ42 were detectable in many brain regions of Tg2576 mice at just 2 months of age using ELISA^16^, and similar results were found at 3–4 months of age by others^15^. Thus, soluble forms of Aβ are present at very early ages, and may be sufficient to produce increased excitability because soluble Aβ increases neuronal activity at very low concentrations^44^. Furthermore, adding just picomolar amounts of Aβ to a hippocampal slice increases LTP^47^. Interestingly, higher concentrations decreased LTP in the same study^47^, suggesting that the earliest elevations in Aβ in Tg2576 mice are accompanied by increased excitability and after Aβ levels rise further, excitability may decrease. Notably, after β-amyloid deposition (in a different mouse model) there were small areas of hyperexcitability adjacent to β-amyloid plaque^48^. Therefore, hyperexcitability does not appear to uniformly decline as more and more plaque accumulates; instead, hyperexcitability appears to vary. The instability of aberrant excitability across the brain is presumably a reason why IIS frequency became variable at the ages when β-amyloid plaque develops in Tg2576 mice. Compensatory responses are also likely, as previously suggested^49^.

In this context it is interesting to consider that one of the regions where Aβ40 and Aβ42 were detected in our earlier study of 2 month-old Tg2576 mice was the brainstem^16^, the location of cholinergic neurons which have been implicated in sleep^22^. Because cholinergic neurons are vulnerable to Aβ^50^,^51^,^52^ and low Aβ concentrations increase neuronal excitability (as discussed above), one would predict that cholinergic neurons would increase their excitability as Aβ levels become slightly elevated above normal. Because muscarinic agonists can produce spontaneous discharges and even seizures^23^, cholinergic neurons that increase their excitability in response to low levels of Aβ could potentially contribute to hyperexcitability. One would predict that such IIS would emerge during a time when ACh release is normally high, i.e. REM sleep^26^. With age, as Aβ increases further, IIS may become variable (as discussed above). ACh levels may continue to rise, but cholinergic neurons may lose function, complicating the situation. The idea that cholinergic neuron function declines is suggested by degenerating cholinergic fibers in 19 month-old Tg2576 mice^53^,^54^. The observation that hyperexcitability of a subset of neurons precedes their dysfunction or deterioration has been suggested before - in rodent models of amyotrophic lateral sclerosis (ALS), where motorneurons exhibit an early hyperexcitable stage^55^, and it has been hypothesized that the early hyperexcitability contributes to later motorneuron degeneration^56^.

Notably, IIS were recorded synchronously from both hemispheres using cortical and hippocampal recording sites. Therefore, IIS reflect synchronized activity in large areas of the neocortex and hippocampus. Cholinergic pathways are good candidates to entrain widespread areas of the cortex and hippocampus because of their diffuse projections. One that is relevant because of its role in sleep is the cholinergic projection of the lateral dorsal tegmentum and pedunculopontine nuclei in the brainstem which innervate areas of the midbrain reticular formation, thalamus, and the forebrain. Another cholinergic projection - one that has been extensively implicated in AD - is from the forebrain cholinergic nuclei (including the MS), with neurons that also have widespread projections to cortical regions. Our data support the idea that these cholinergic projections are important because increased ChAT-ir was evident in the areas corresponding to the axon terminals at only 4 months of age and there was an increase in c-fos-ir in an area corresponding to the cell bodies, the MS. Our findings are consistent with a prior study showing greater vesicular acetylcholine transporter (VAChT)-labeled cholinergic boutons in frontal cortex of Tg2576 mice at 8 months of age^51^. Interestingly, there was variability in subregions, which we found with ChAT-ir also (Fig. 5; Supplementary Fig. 9).

Based on these considerations, we suggest that IIS occur early in life and are rare initially. As IIS become significant in the first months of life, an increase in cholinergic tone contributes to IIS, possibly caused by initial elevations in Aβ. IIS are initially specific to REM, presumably because of the increased release of ACh during this sleep stage. By divergent projections to hippocampus and cortical regions, ACh could facilitate synchronized discharges in these locations – manifested as an IIS. Muscarinic receptors appeared to be important to IIS, but additional mechanisms are likely to contribute based on pharmacology and data from APP51 mice. Later in life as Aβ levels and other abnormalities emerge, the data suggest that IIS become variable rather than continuing to escalate (Fig. 7).

### IIS and hyperexcitability

In the preceding discussion we consider IIS to reflect abnormal excitability, consistent with the underlying cellular correlate of IIS, a sudden and synchronous depolarization in principal neurons (paroxysmal depolarization shift^57^). Notably, IIS in Tg2576 mice at 5 weeks of age do not appear to be “interictal” since seizures were not detected. Therefore, this aberrant form of excitability appears to be a particular subtype of IIS, so-called “preictal” IIS, which occur in the weeks before the first seizure in animals that develop epilepsy^58^,^59^. The idea that the 5 week-old Tg2576 mouse is at a stage of life where seizures are imminent but not yet present (i.e., preictal) is consistent with a lower threshold for pentylenetetrazol-induced seizures in 6 week-old Tg2576 mice compared to WT mice^14^.

As Tg2576 mice aged and developed seizures, IIS were interictal (no longer preictal). In contrast to the idea that IIS are a reflection of hyperexcitability, interictal IIS in epilepsy may abort or inhibit seizures^60^ possibly by inhibiting the pathological mechanisms that produce a severe seizure. This inhibitory effect of IIS may have been strong in Tg2576 mice, because we found that spontaneous seizures were rare - even when mice had a seizure, frequency was only 1 per 2 weeks.

Regardless of the consequences of IIS on spontaneous seizures, there is good reason to believe that IIS adversely affected cognition, because their frequency became most robust at the age when behavioral impairments have been shown to emerge, (approximately 3 months of age^15^,^16^). IIS are likely to disrupt cognition based on previous studies of IIS in epileptic rats and humans^61^,^62^. This idea is consistent with other studies^6^ showing that memory was improved after epileptiform activity was reduced by levetiracetam. In another study that examined hyperactivity by fMRI, patients with mild cognitive impairment had improved memory after levetiracetam was used to decrease the hyperactivity^5^. Thus, IIS and other types of hyperexcitability in mouse models and in humans could be a characteristic that, if treated, would improve symptoms, which has been suggested before^2^,^3^.

### IIS, sleep and progressive Aβ neuropathology

IIS occurred in sleep or quiet wakefulness, consistent with other mouse models of AD that have described hyperexcitability when mice are motionless^20^. These findings have significant implications because it has been suggested that sleep (and quiet wakefulness) subserve important functions related to memory consolidation^63^,^64^, which IIS are likely to disturb (as discussed above). Whether IIS directly cause memory disturbance in Tg2576 mice is only one possible conclusion, however. Alternatively, other abnormalities cause memory dysfunction and IIS may contribute but are not causal. These two possibilities are difficult to dissociate because the effects of IIS and other early disturbances appear to be interrelated. For example, Aβ levels increase with wakefulness but decrease during sleep in Tg2576 mice^65^, presumably because normal sleep clears the brain of Aβ^66^. Increased β-amyloid leads to a disturbed sleep-wake cycle and reduces the sleep-related fluctuation of Aβ in the brain^67^. Furthermore, hyperexcitability in rodents leads to increased release of Aβ into the extracellular milieu^68^. Therefore, IIS might increase Aβ release and simultaneously impair the processes during sleep that clear Aβ. This view would be consistent with the idea that IIS cause the progression of AD neuropathology. Alternatively, very low concentrations of Aβ could disrupt the normal activity levels of cholinergic neurons (as discussed above), which in turn could cause IIS and sleep disturbances. This perspective, where Aβ is the cause of pathology, would be more consistent with current views.

In summary, IIS may cause impairments that develop at later ages such as memory impairment, sleep disturbances and even increased Aβ levels. However, it seems more likely that IIS interact in complex ways with Aβ levels and sleep disturbances, explaining IIS variability.

### Implications

Our goal was to study the early changes in the brain that led to hyperexcitability and their potential implications. We found that a surprisingly early sign of hyperexcitability are IIS in REM sleep, possibly “preictal” IIS. The inappropriate synchronization of neural networks caused by IIS is likely to contribute to the initial changes in the brain that ultimately become interrelated and complex, causing variable IIS frequency and memory impairment. Thus, IIS may be an important early step and the results here suggest an explanation for the variable nature of hyperexcitability that is consistent with diverse clinical observations regarding hyperexcitability. These findings also raise the possibility that IIS in sleep may be a useful biomarker in detecting the earliest phases of AD.

## Methods

### Animals

Experimental procedures were approved by the Institutional Animal Care and Use Committee at The Nathan Kline Institute and experiments were conducted in accordance with approved guidelines. Mice which express human APP_695_ with the Swedish (Lys670Arg, Met671Leu) mutations driven by the hamster prion protein promoter^8^ were bred from male heterozygous Tg2576 and female non-transgenic mice (C57BL6/SJL F1 hybrid). APP51 mice (WT-APP_751_; C57BL6 background) were generously provided by Dr. Paul Mathews. These mice overexpress the 751 isoform of human WT-APP without familial mutations of AD driven by the Thy-1.2 promoter^21^. Food (Purina 5001, W.F. Fisher) and water were available *ad libitum*.

### Surgery

Surgical and implantation procedures were performed as previously described^17^. Mice were anesthetized with chloral hydrate (55 mg/kg, i.p.) and placed in a stereotaxic apparatus (David Kopf). After exposing the skull, 4 electrodes were positioned: 1) a monopolar 90 μm-diameter stainless steel wire (California Fine Wire Co.) in each dorsal hippocampus (2.0 mm posterior to Bregma, 2.5 mm lateral to the midline, 2.0 mm deep), 2) two subdural screw electrodes (2.5 mm diameter screws, Pinnacle Technologies), one over left frontal cortex (0.5 mm posterior to Bregma, 1.5 mm lateral to the midline) and one over right occipital cortex (3.5 mm posterior to Bregma, 2.0 mm lateral to the midline). An epidural screw electrode was placed above the cerebellum at the midline to serve as reference and a subdural screw electrode over the olfactory bulb was used as the ground. An 8-pin connector (Millmax) was centered over the skull with dental cement (Dentsply) and the animal was placed in its home cage on top of a heating pad (37 °C; Harvard Apparatus) until fully ambulatory.

## Video-EEG

### Data Acquisition

Mice were housed individually for at least a week after surgery in the room where EEG was conducted so that they would acclimate to the recording environment. Recordings were performed in a 21 cm × 19 cm cage similar to a standard mouse cage with a multichannel commutator (Pinnacle Technologies) to allow freedom of movement.

Signals were acquired at 2000 Hz sampling rate and bandpass filtered at 0.5–200 Hz (Pinnacle). Simultaneous video was recorded continuously at 10 frames per second (synchronized with the EEG record) during both light and dark periods using an infrared LED camera with Sirenia Video Acquisition software (Pinnacle).

### Data Analysis

Data analysis was performed using Spike2 (Cambridge Electronic Design) and MATLAB (MathWorks) with the FieldTrip toolbox^69^, the Statistics/Signal Processing toolboxes, and the MLIB package [Mathworks File Exchange ID #37339].

#### Detection of spikes

IIS were defined as transient (≤75 milliseconds), large (7 zscores above and below the mean of the baseline of each channel) amplitude events occurring in all 4 recording channels. An amplitude threshold of 7 zscores was selected because this criterion most closely approximated IIS detection performed by manual review (Supplementary Fig. 11e). Selection of the 7-zscore threshold, a relatively high threshold, also excluded putative cortical sleep spindles and hippocampal sharp waves because they were much smaller in amplitude (Supplementary Fig. 3a). IIS onset was defined as the time of the peak of the first IIS in any channel and was detected using an iterative template-based approach (Supplementary Fig. 11c-e). All IIS were captured by setting a defined period of time (waveform window) that captured the entire IIS (50 milliseconds before the peak and 150 milliseconds after the peak or a total of 200 milliseconds). Templates were refined by conducting principal component analysis with k-means clustering (5–10 clusters per recording for both IIS and artifacts). The first 10 principal components were sufficient to distinguish IIS waveform from artifacts (Supplementary Fig. 11c). Artifacts were confirmed by manual review of video-EEG and were associated with scratching or other movements that disrupted the cable/headstage.

#### Peri-event time histogram

PETHs were constructed using standard methods with custom-written scripts in MATLAB using the peak of the IIS and the onset of the behavioral state (e.g., REM). Raw histograms were smoothed with a Gaussian kernel (defined by a 3 second bandwidth) and averaged across animals of the same genotype/age.

#### Detection of behavioral states

Video-EEG was analyzed in 10 second-long periods to characterize behavioral states^70^. Behavioral states (active wakefulness, quiet wakefulness, NREM and REM sleep) were classified primarily on the basis of these criteria (Supplementary Fig. 2):

1. Ratio of theta to delta power (θ, 5–10 Hz; δ, 0.5–4 Hz)^71^ using the frontal cortical recording site.
2. Wideband power (0.5–200 Hz) of the frontal cortical recording.
3. Movement, detected by positional tracking and confirmed by simultaneous manual review of video.

Animal position during video-EEG recordings was tracked using the Kalman filter, image segmentation, and blob detection technique (Sirenia, Pinnacle). The raw velocity vector was smoothed with a Gaussian kernel (bandwidth = 500 milliseconds).

REM sleep was defined by a high ratio of theta/delta power (>2.5 for 10 second epochs, Supplementary Fig. 2), and little or no movement of the body (based on both positional tracking and manual review as described above). In addition, a criterion for REM sleep was that the prior behavioral state was NREM sleep (which is the normal pattern for sleep in rodents). REM sleep segments separated by less than 3 seconds were merged because these were periods when small twitches or slight posture changes appeared to interrupt an otherwise continuous period of REM sleep.

If movement was minimal, but other criteria for REM were not met, the behavioral state was classified as NREM sleep or quiet wakefulness. NREM was discriminated from quiet wakefulness based on the zscored wideband power, power in the delta band and presence of putative spindles (Supplementary Figs 2 and 3a). Thus, NREM sleep showed a lower ratio of theta/delta power (<2.5), and greater wideband power (>1 zscore for at least 3 second) than quiet wakefulness. Sleep episodes were confirmed manually by reviewing video and finding that mice had a curled, supine position.

Epochs with relatively low delta power, low wideband power, and minimal movement were designated as quiet wakefulness. All epochs with movement for >3 seconds were classified as active wakefulness and included exploration/walking, grooming, sniffing and consummatory behavior (eating/drinking). All spectral thresholds were verified manually for each recording. After manual staging of behavioral states, we implemented a Random forest supervised learning algorithm^72^ to reveal additional differences in each brain state to allow for automated classification (Suppl. Fig. 3b–d).

#### Fragmentation of sleep

A Sleep Fragmentation Index (SFI)^73^ was modified for rodents. SFI is defined as the number of transitions from NREM or REM and number of brief arousals within a sleep episode. The arousals were considered brief if they lasted <3 seconds and the sleep epoch was considered to end if the arousals lasted >3 seconds. The lower limit of 3 seconds was chosen because intermittent twitches and posture changes during sleep appeared to occur in all mice (regardless of genotype) but if they continued for more than 3 seconds sleep appeared to end (e.g. the animal began to explore). A sleep episode was defined as a period of time beginning with NREM and terminating with either a REM or NREM sleep epoch with the total duration >5 minutes.

#### Detection of seizures

Seizures were detected offline based on the wideband power in the EEG, using a threshold of 4 zscores for a minimum of 5 seconds in any single recording channel (Hanning window, 2 second non-overlapping window). These criteria were chosen because they easily discriminated seizures from other behaviors not only in Tg2576 mice but also other mice with seizures using the same recording system^17^. The video and EEG record was also manually reviewed, which confirmed the analysis by wideband power. All seizures were accompanied by severe convulsive behavioral manifestations (i.e., Racine scale 4–5^74^). For the 2 week-long continuous recordings, there was an interruption of the continuous recording for two days (in 2 of the 8 mice recorded > 8 months of age), so an additional two days was added at the end of the 2 weeks.

#### Spectral analysis

Data were downsampled to 1000 Hz and cut into 10 second-long segments. Segments that were contaminated by mechanical noise or movement/cable artifacts were discarded. Power spectra were computed using a single Hann taper Fast Fourier Transform (FFT) approach. Raw FFT power spectra were then standardized by zscore in the frequency domain using the mean and standard deviation of the EEG during periods of quiet wakefulness within a given recording session. For analysis of peak theta frequency, power spectra between 5 and10 Hz (the typical frequency range for theta in mice) were computed during REM periods. Distributions of the peak theta frequency were calculated from the local maxima estimation of each power spectrum in all REM sleep epochs during a 24 hour-long recording for every animal.

#### Time-frequency analysis

Data were downsampled to 1000 Hz and the time-frequency representation was computed using a wavelet approach. We convolved the data with a complex Morlet wavelet (7-cycle density per frequency). Raw wavelet power spectra were then standardized by zscore in the frequency domain using the mean and standard deviation of wavelet power from periods of quiet wakefulness within a given recording session.

### ChAT and c-fos immunohistochemistry

#### Perfusion-fixation and preparation of tissue

Mice were deeply anesthetized by isoflurane inhalation followed by urethane (2.5 g/kg i.p.) and transcardially perfused with a peristaltic pump (Minipuls 1) using 10 ml of 0.9% NaCl in double-distilled water (ddH_2_0) followed by 40 ml of 4% paraformaldehyde in 0.1 M phosphate buffer (PB; pH 7.4). The brains were removed immediately and post-fixed for 24 hours in 4% paraformaldehyde in 0.1 M PB at 4 ^o^C. After post-fixation, brains were hemisected and one hemisphere was cut in the horizontal plane into 50 μm-thick sections using a vibratome (Vibratome Co.). Sections were collected sequentially so that when choosing sections to process it was possible to compare equivalent dorso-ventral levels across animals and the sections were equidistant (300 μm apart).

#### Immunohistochemistry

A goat polyclonal antibody against ChAT was raised against human placental ChAT, affinity purified and confirmed by Western blot to label a band similar to the molecular weight of ChAT^75^ (AB144P, Millipore). For c-fos, a goat polyclonal antibody (sc-52-G, Santa Cruz) was raised against the N-terminus peptide of human c-fos (amino acids 3–16). Western blot was used to show that the antibody recognized a 62 kD protein corresponding to the molecular weight of c-fos (manufacturer’s datasheet).

Sections from WT and Tg2576 were processed together. Methods similar to those used previously were used for washing and preparation of the tissue^16^,^76^. Modifications to these methods were as follows: sections were blocked (10% normal horse serum; Vector Laboratories), and incubated for 24 hours in the primary antiserum (ChAT, 1:1,000; c-fos, 1:10,000) followed by secondary antibody (horse anti-goat; 1:400; Vector Laboratories), and incubation in avidin-biotin-horseradish peroxidase complex (Vectastain Elite Kit, Vector Laboratories). Sections were subsequently incubated in 0.022% DAB, 0.2% NH_4_Cl, 0.1% glucose oxidase, 0.8% D(+)-glucose and 2% NiCl_2_ in 0.1 M Tris buffer.

#### ChAT and c-fos analysis

Sections were examined using a brightfield microscope (BX61, Olympus) and photographed using a digital camera (RET 2000 R-F-CLR-12, Q Imaging). For the hippocampal ChAT analyses shown in Fig. 5, four sections were chosen from each animal using similar dorso-ventral levels, and were photographed using the same microscope and software settings. Animals that are listed as 1 month-old were 0.9 ± 0.05 (range 0.9–1.3) months-old; 4 month-old mice were 3.7 ± 0.08 (range 3.3–4.5); 14 month-old mice were 14.4 ± 0.9 (11.4–16.3) months-old. Micrographs were analyzed using Image J (National Institutes of Health) by conversion to grayscale and then measuring mean pixel density (MPD; 0–255, with 0 black and 255 white) within a fixed areas (the hilus, CA1 stratum radiatum and CA3; Fig. 5b). To correct for background staining, the MPD of an area of white matter without ChAT labeling was analyzed (Fig. 5b). For analysis of c-fos, two sections containing the MS and LS were chosen from each animal. In an area of 600 μm^2^ for each region, c-fos-ir cells were thresholded in image J, counted and cell density was calculated using the same methods used for counting ChAT-ir cells (see supplementary information for details).

### Drug Treatment

All drugs were made on the day of use, dissolved in 0.9% NaCl, and injected i.p. The dose of atropine sulfate (Sigma Aldrich, 50 mg/kg) was based on its ability to reduce theta in rodents^77^,^78^. The dose of levetiracetam (Cayman Chemicals, 200 mg/kg) was the same as the dose used to block epileptiform activity in an APPSwe/Ind mouse model of AD (“J20”)^6^. Mecamylamine (Tocris Bioscience), a nonselective nicotinic receptor antagonist, was used at a dose (10 mg/kg) that was effective against epileptiform activity in a previous rodent study^79^. Methyllycaconitine (Sigma Aldrich), an α7nicotinic receptor antagonist, was used at 10 mg/kg where it blocked seizures in mice^80^. The dose of the cholinesterase inhibitor donepezil (Eisai Co., 4 mg/kg) was chosen based on use in Tg2576 mice^19^,^81^. We excluded three mice due to the absence of sleep in the first 4 hours after drug injection. Two of these mice received atropine and one received donepezil. In three mice, 3-day sessions were tested 2 or 3 times in succession, so that mice were administered atropine, levetiracetam and donepezil. There was a 2 week interval between consecutive 3-day sessions to ensure the initial drug treatment session did not influence the next session.

### Statistical Analysis

All data are reported as mean ± S.E.M and the *p* criterion was 0.05. Statistical comparisons were performed in MATLAB. For comparison of two means, either paired t-tests or two sample t-tests were used. For comparison of more than 2 groups, we used one-way, two-way, or three-way ANOVA with Tukey-Kramer’s post hoc test. Repeated measures ANOVA followed by the Tukey-Kramer post hoc test was used to analyze sequential EEG recordings. For data sets with zero values or a failed Lillefors test of normality (p < 0.05), non-parametric tests were used (Mann-Whitney for 2 groups and Kruskal-Wallis for > 2 groups with Dunn-Sidak post hoc tests).

## Additional Information

**How to cite this article**: Kam, K. *et al*. Interictal spikes during sleep are an early defect in the Tg2576 mouse model of β-amyloid neuropathology. *Sci. Rep.* **6**, 20119; doi: 10.1038/srep20119 (2016).

## Supplementary Material

Supplementary Information
