# Supplementary material for: Interictal spikes during sleep are an early defect in the Tg2576 mouse model of β-amyloid neuropathology
Source: Sci Rep. 2016 Jan 28;6:20119. doi: 10.1038/srep20119 (PMC4730189; doi:10.1038/srep20119)
Supplement: Supplementary Information [file srep20119-s1.doc]

**SUPPLEMENTARY INFORMATION for**

### Interictal spikes during sleep are an early defect in the Tg2576 mouse model of β-amyloid neuropathology

Korey Kam^1,4^, Áine M. Duffy^1,2^, Jillian Moretto^1^, John J. LaFrancois^1^ and Helen E. Scharfman^1,2,3*^

^1^The Nathan Kline Institute for Psychiatric Research

Center for Dementia Research

Orangeburg, NY 10962

^2^Department of Physiology and Neuroscience

New York University Langone Medical Center

New York, NY 10016

^3^Department of Child and Adolescent Psychiatry and Psychiatry

New York University Langone Medical Center

New York, NY 10016

^4^Graduate Program in Physiology and Neuroscience

New York University Langone Medical Center

New York, NY 10016

**Supplemental Methods (Immunohistochemistry)**

To measure cell density, cells were counted within a rectangular region of interest (ROI) placed over the dorsal striatum or midline (for medial septum). Density was defined as (cell number)/(ROI area). Similarities of calibration measurements suggested shrinkage was similar in both groups so no correction for shrinkage was made. The ROI was selected so that it was large enough to contain >5 cell bodies (ChAT: dorsal striatum 500 µm^2^, medial septum 350 µm^2^; c-fos: medial septum and lateral septum, 600 µm^2^). For each animal, values were obtained from 4 sections (striatum) or 2-3 sections (medial septum) and averaged. Counts were made using ImageJ (National Institutes of Health), with cells above a threshold level of immunoreactivity included and cells below the threshold rejected; thresholds were set beforehand so that ChAT-ir or c-fos-ir cells were above and background was below threshold (Lee et al., 2012, Duffy et al., 2013). When overlapping cells were detected, they were counted manually from the original slide so that multiple focal planes could be used to discriminate cell bodies. Manual counts were made in 2 mice to confirm that cell counts using thresholding were similar to manual counts (<5% different).

MPD for striatum and medial septum were measured from ROIs that were used for cell counting. MPD for retrosplenial cortex (RSC) used a rectangular ROI that extended from the pial surface into the cortex by 400 µm, so it encompassed the superficial layers where ChAT-ir was greatest. Micrographs used for the measurements were obtained with the same camera settings. MPD was calculated using ImageJ, as for MPD in Figure 5.

Reference:

Lee H, Dvorak D, Kao HY, Duffy AM, Scharfman HE, Fenton AA (2012) Early cognitive experience prevents adult deficits in a neurodevelopmental schizophrenia model. Neuron. 75:714-24.

Duffy AM, Schaner MJ, Chin J, Scharfman HE (2013) Expression of c-fos in hilar mossy cells of the denate gyrus *in vivo*. Hippocampus. 23: 649-655.

**Supplementary Table 1** | **Longitudinal recordings of Tg2576 WT and APP51 mice.**

Individual animals are listed with the IIS frequency (mean IIS/hour) for an entire 24 hour-long recording session.

|  | Age (months) | | | | | | | | | |
| --- | --- | --- | --- | --- | --- | --- | --- | --- | --- | --- |
| Animal ID# | 1.2 | 2.0 | 3.0 | 4.0 | 5.0 | 6.0 | 7.0 | 8.0 | 9.0 | >7.0 |
| Tg2576 WT |  |  |  |  |  |  |  |  |  |  |
| 024 | 0 | 0 | - | - | 0 | - | - | - | - | - |
| 025 | 0 | 0 | 0 | - | 0 | - | 0 | - | - | - |
| 048 | 0 | 0 | 0 | 0 | - | - | 0 | - | - | - |
| 030 | 0 | 0 | 0 | - | 0 | 0 | - | - | - | - |
| 031 | 0 | 0 | 0 | - | 0 | - | - | - | - | - |
| 021 | 0 | 0 | - | 0 | - | 0 | - | - | - | 0 (21 mon) |
| 052 | 0 | - | - | - | - | - | - | - | - | - |
| 413 | 0 | 0 | 0 | 0 | 0 | 0 | 0 | - | - | - |
| 419 | 0 | 0 | 0 | 0 | 0 | 0 | - | - | - | - |
| 269 | - | - | - | - | 0 | 0 | 0 | - | - | - |
| 271 | - | - | - | - | 0 | - | 0 | - | - | - |
| 063 | - | - | - | - | - | - | - | - | - | 0 (23 mon) |
| 077 | - | - | - | - | - | - | - | - | 0 | - |
| 078 | - | - | - | - | - | - | - | - | 0 | - |
| 079 | - | - | - | - | - | - | - | - | 0 | - |
| 080 | - | - | - | - | - | - | - | - | 0 | - |
| 081 | - | - | - | - | - | - | - | - | 0 | - |
|  |  |  |  |  |  |  |  |  |  |  |
| APP51 |  |  |  |  |  |  |  |  |  |  |
| 061 | 0 | 0 | 0 | 0 | 0 | 0 | 0 | - | - | - |
| 066 | 0 | 0 | 0 | 0 | 0 | 0 | 0 | - | - | - |
| 067 | 0 | 0 | 0 | 4.67 | 10.17 | 8.83 | 19.88 | - | - | - |
| 094 | - | - | - | - | - | - | 3.29 | - | - | - |
| 095 | - | - | - | - | - | - | 0.71 | - | - | - |
|  |  |  |  |  |  |  |  |  |  |  |


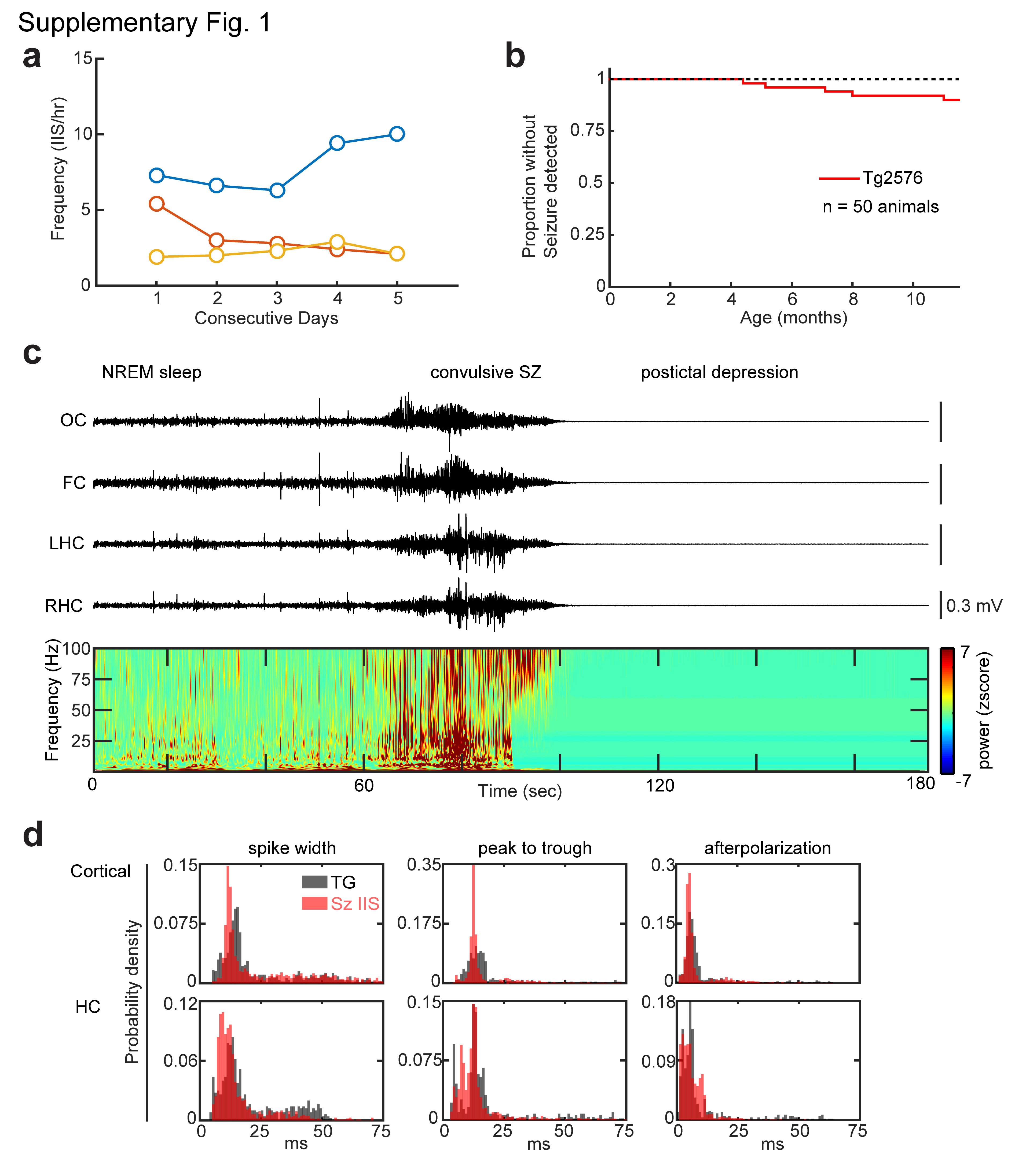


Suppl. Fig. 1 | Additional characteristics of IIS and seizures in Tg2576 mice.

1. A 5 day-long recording period for 3 mice at 5 weeks of age showed no significant effect of time on IIS frequency (repeated measures ANOVA, F(4,14)2.363, p=0.367).
2. Kaplan-Meier plot of seizures in 50 Tg2576 mice that were recorded for one or more 24 hour-long periods. Five out of 50 mice displayed spontaneous seizures.
3. Top: A representative example of an EEG recording of a seizure. The seizure began in NREM sleep without IIS (Figure 1) and the animal had a stage 5 convulsive seizure (convulsive SZ), which was common to all seizures.
4. Histograms illustrate the similarity of IIS in mice with and without seizures. The results compare IIS spike width, peak to trough duration, and afterpolarization duration from 5 week-old Tg2576 mice (n=7) without seizures and IIS of older Tg2576 mice with seizures (n=5, 7.9 ± 2.3 months of age).


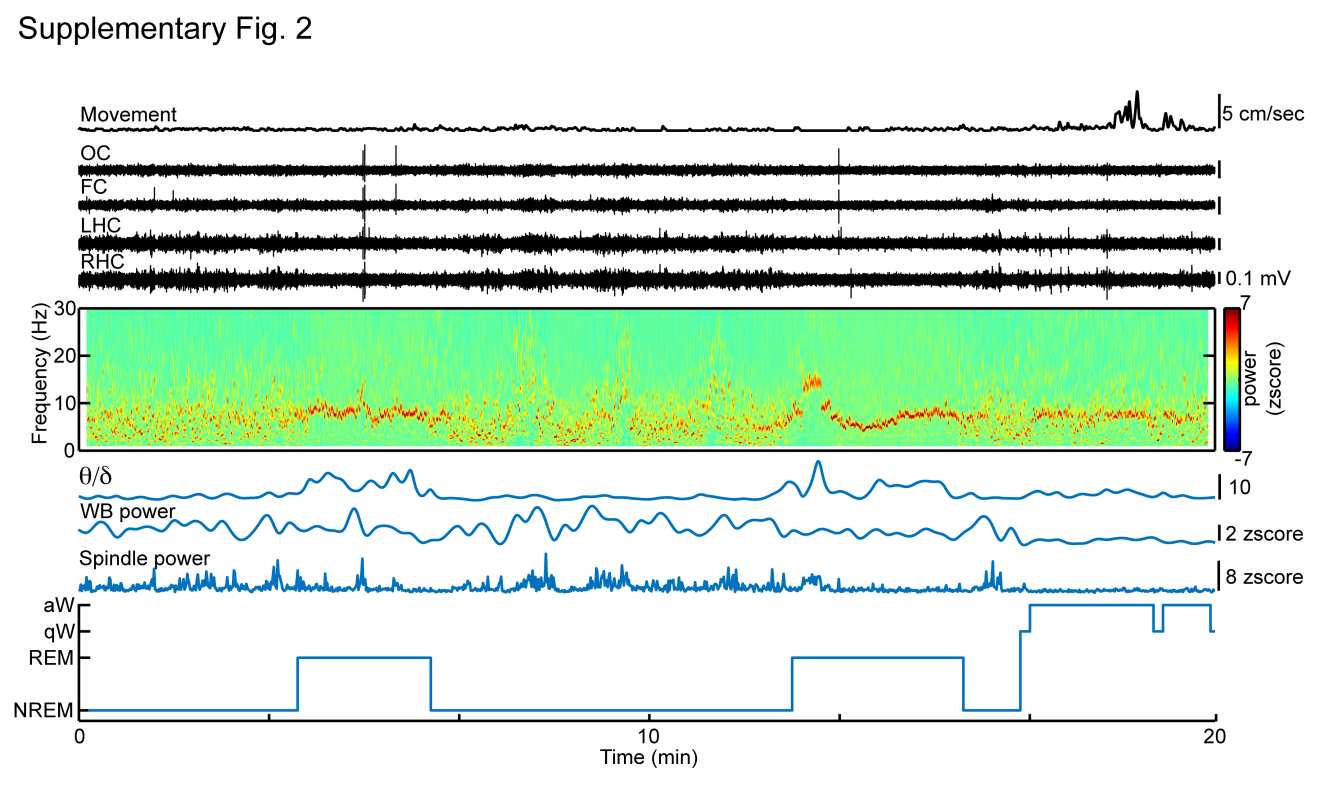


Suppl. Fig. 2 | Behavioral state detection.

An example of behavioral state detection (extended data from Figure 2b). The criteria included (from Top to Bottom) movement, EEG recordings (4 recording sites, abbreviations as in Fig. 1), spectrogram, theta/delta (θ/δ) ratio, and wideband (WB) spectral power. Spindle power (10-18 Hz) was also used to define putative spindles. At the bottom the behavioral states are shown, based on the criteria depicted above. aW = active wakefulness; qW = quiet wakefulness.


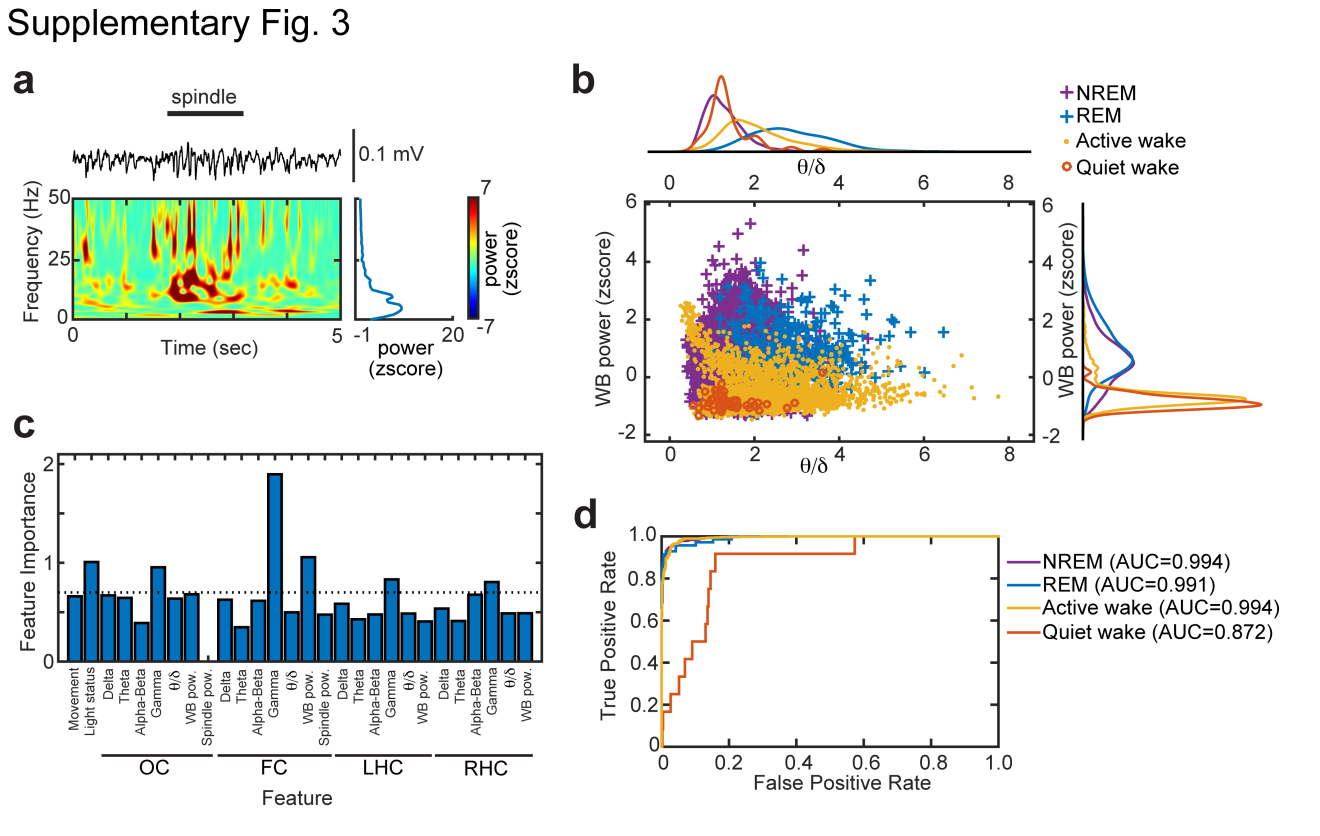


Suppl. Fig. 3 | Behavioral state assessment.

1. A representative EEG trace during NREM sleep that includes a putative cortical spindle, identified by the spectrogram (bottom left) corresponding to the EEG and power spectrum (bottom right; see Methods).
2. Scatter and probability plots show the distribution of behavioral states in a 24 hour-long recording by θ/δ and the wideband spectral power marginals.
3. The features used to classify behavioral state with a Random forest classifier are plotted with relative feature importance (“out-of-bag error”) for a single 24 hour-long recording.
4. Receiver operating characteristic (ROC) curve demonstrates accuracy of behavioral state detection based on the area under the curve (AUC). True positive rate vs. false positive rate of the classifier output is plotted for each behavioral state. The classifier was most accurate in predicting REM and NREM sleep states and least accurate for periods of quiet wakefulness.


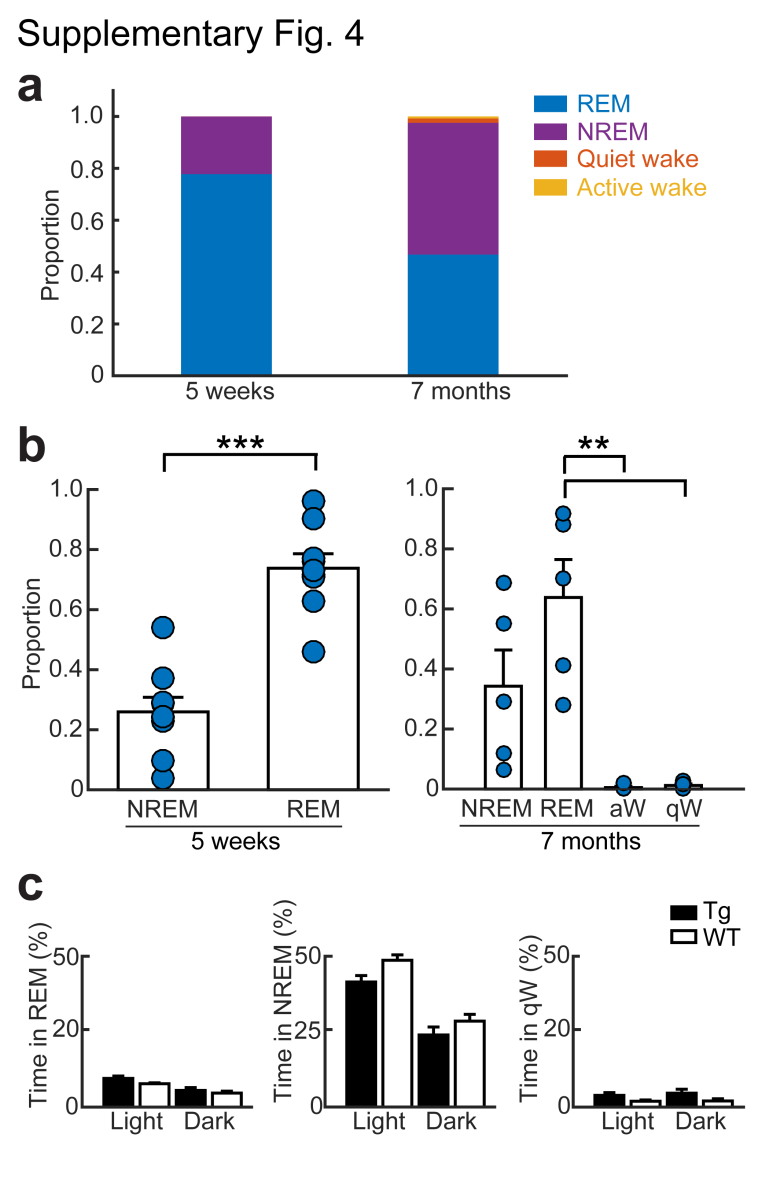


Suppl. Fig. 4 | Additional analysis of IIS in relation to behavioral state.

1. Proportion of IIS in each behavioral state at 5 weeks (n=9) and 7 months of age (n=5) in Tg2576 mice. At 5 weeks of age, IIS predominate in REM sleep (blue). At 7 months of age, IIS are more abundant in NREM sleep (purple). At both ages, IIS in quiet wakefulness and active wakefulness are rare.
2. Left: The proportion of IIS in REM sleep was significantly greater than the proportion of IIS in NREM sleep at 5 weeks of age (n=9 mice, Student’s t-test, p<0.0001). Right: Proportion of IIS in all behavioral states at 7 months of age (n=5 per group). REM and NREM sleep were not different, but REM was significantly different from each wakeful state (Kruskal-Wallis test, H(19)60, p=0.002; post-hoc tests, p<0.05). IIS in wakefulness (quiet) emerged at 7 months of age but were rare.
3. Left: Time in REM sleep is shown for the light and dark periods of the light/dark cycle. Time in REM was defined as the sum of all REM epochs divided by the length of the light or dark cycle (12 hours) and is expressed as a percent. Tg2576 mice (n=9) and WT mice (n=5) were similar at 5 weeks of age, with no effect of genotype (two-way ANOVA, F(1,27)2.173, p=0.153). As expected because mice sleep during the light period, there was more REM in the light period for all mice (effect of light vs. dark period, F(1,27)15.643, p=0.0005). Center: Time in NREM sleep is shown for the same animals. There were differences in all animals depending on the light or dark period (two-way ANOVA, F(1,27)48.956, p<0.0001) but no differences between Tg2576 and WT mice (post-hoc tests, p>0.05). Right: Time in quiet wakefulness for the same mice showed no effects of genotype (two-way ANOVA, F(1,27)3.749, p=0.065) or light status (F(1,27)0.120, p=0.732).


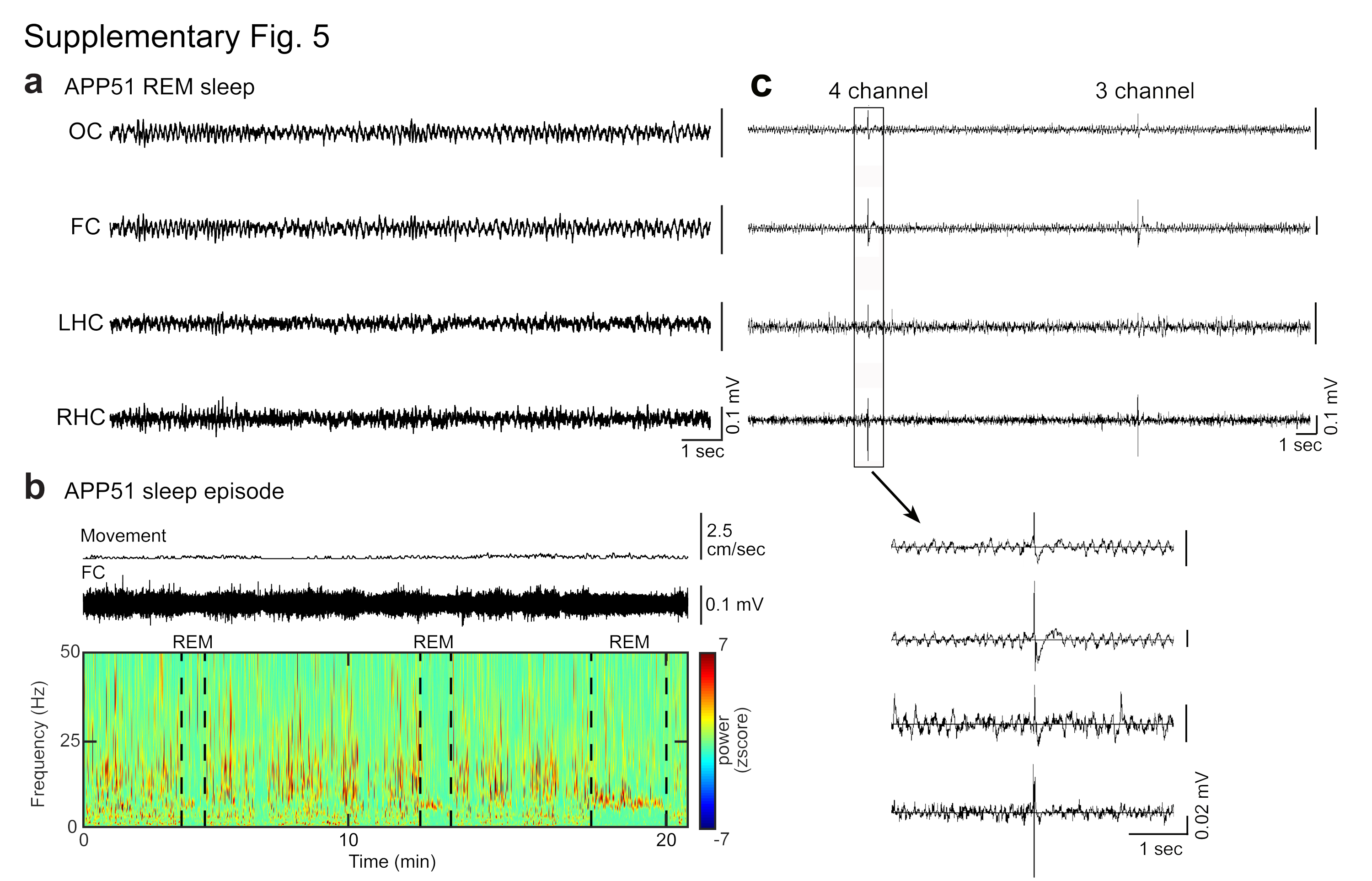


Suppl. Fig. 5 | Representative recordings from APP51 mice show no IIS at 5 weeks of age.

1. An example of an EEG recording during REM sleep from a 5 week-old APP51 mouse (WT-APP overexpression without a familial AD mutation) shows no IIS and normal theta oscillations.
2. A representative 20 minute-long sleep episode shows that IIS did not occur in REM or NREM sleep.
3. An example of two IIS recorded during REM sleep from a 7 month-old APP51 mouse. The IIS surrounded by the box is expanded at the bottom. This IIS met all criteria used to define IIS in Tg2576 mice. In this APP51 mouse we also found IIS with spike-like transients in only 3 of the 4 channels, and one is shown (it is marked “3 channel”). The left hippocampal recording fails to show any significant transient (i.e., significantly different in amplitude relative to the baseline noise). These 3 channel, IIS-like events were also detected in another APP51 mouse at 7 months of age, where they also occurred during REM sleep. In total, 5 of 5 APP51 mice had 3 or 4 channel IIS (6.20 ± 3.47 IIS/hr).


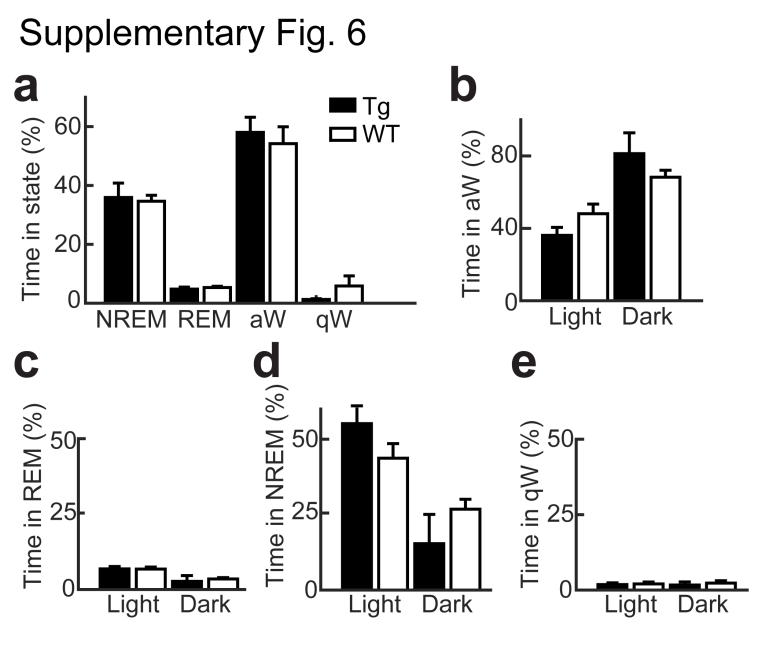


Suppl. Fig. 6 **|** Comparisons of behavioral state in 7 month-old Tg2576 and WT mice.

1. Time spent in each behavioral state per 24 hour-long recording is shown for 7 month-old Tg2576 (n=5) and 7 month-old WT mice (n=4). There were no significant effects of genotype (two-way ANOVA, F(1,39)<0.00001, p=0.999) but there were significant differences in the duration of time animals spent in a given behavioral state, with more time in NREM than REM and more time in active wakefulness than quiet wakefulness (two-way ANOVA, F(3,39)64.451, p<0.00001).
2. The percentage of time spent in active wakefulness is plotted for the light and dark periods. Time in active wakefulness was similar for 7 month-old Tg2576 mice (n=5) and age-matched WT mice (n=4) with no effect of genotype (F(1,19)0.006, p=0.941). However, there was an effect of light status (two-way ANOVA, F(1,19)21.771, p<0.001) consistent with more movement in the dark period, typical of rodents.
3. There was no effect of genotype on the time spent in REM sleep (two-way ANOVA, F(1,19)0.102, p=0.754) but there was an effect of light status (F(1,19)11.167, p=0.004) with more REM in the light period, typical of rodents, who sleep more during the daytime. Same mice as in **c**.
4. There was no effect of genotype on the time spent in NREM sleep (two-way ANOVA, F(1,19)<0.001, p=0.998) but there was an effect of light status (F(1,19) 20.879, p<0.001), similar to **c**. Same mice as in **c**.
5. There was no effect of genotype or light status on time in quiet wakefulness (two-way ANOVA, genotype: F(1,19)2.688, p=0.121; light status: F(1,19)0.647, p=0.433).


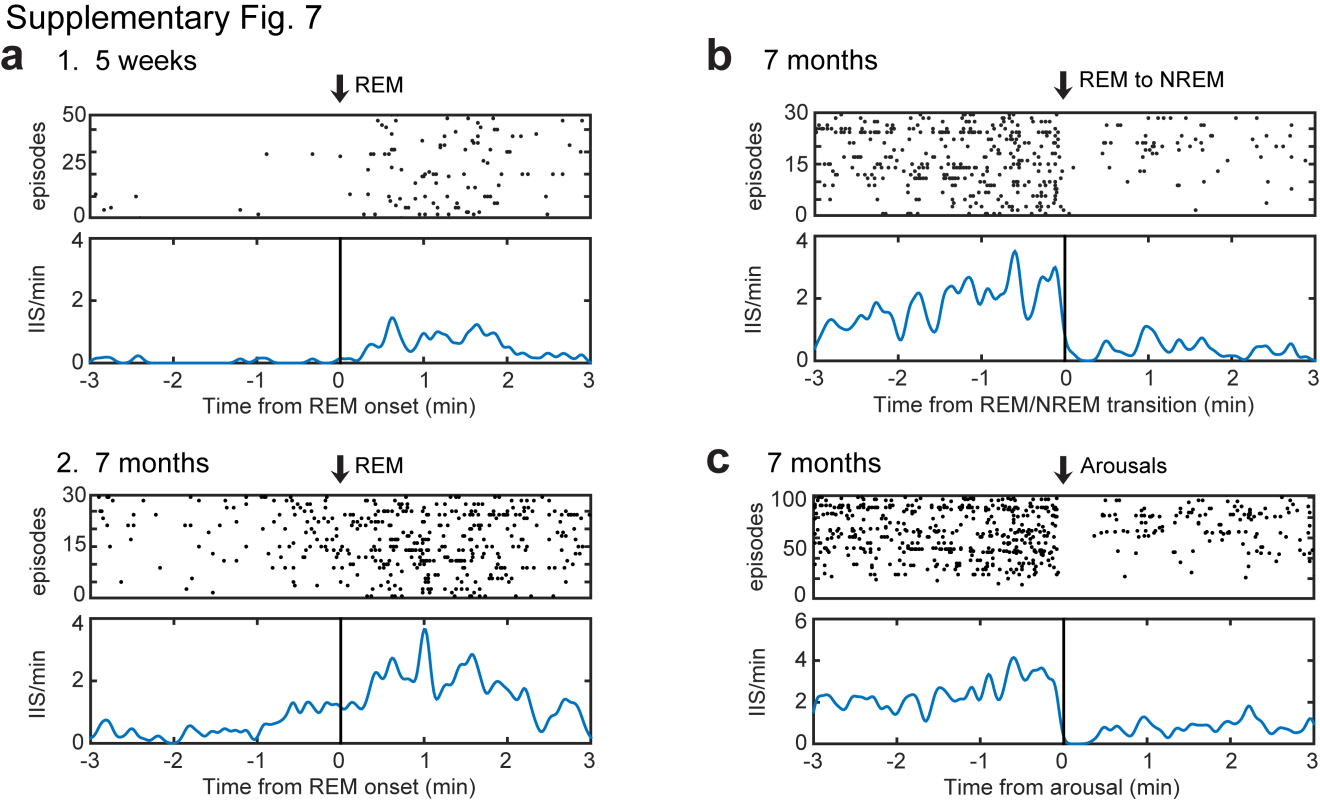


Suppl. Fig. 7 | IIS frequency increases at the start of REM sleep and decreases at the end of REM sleep.

1. A representative raster plot (top) and PETH of IIS (bottom) at the beginning of REM sleep (arrow) for a Tg2576 mouse at 5 weeks of age (1) or 7 months of age (2). IIS frequency increases at the start of REM and this effect is larger in the 7 month-old mouse.
2. A representative raster plot (top) and PETH of IIS (bottom) during a REM/NREM sleep transition for a Tg2576 mouse at 7 months of age. IIS frequency decreased at the transition to NREM from REM sleep. Same animal as shown in **a2**.
3. A representative raster plot (top) and PETH (bottom) of IIS at the time of arousal from sleep in a Tg2576 mouse at 7 months of age. IIS frequency decreased upon arousal from sleep. Same animal as shown in **a2**.


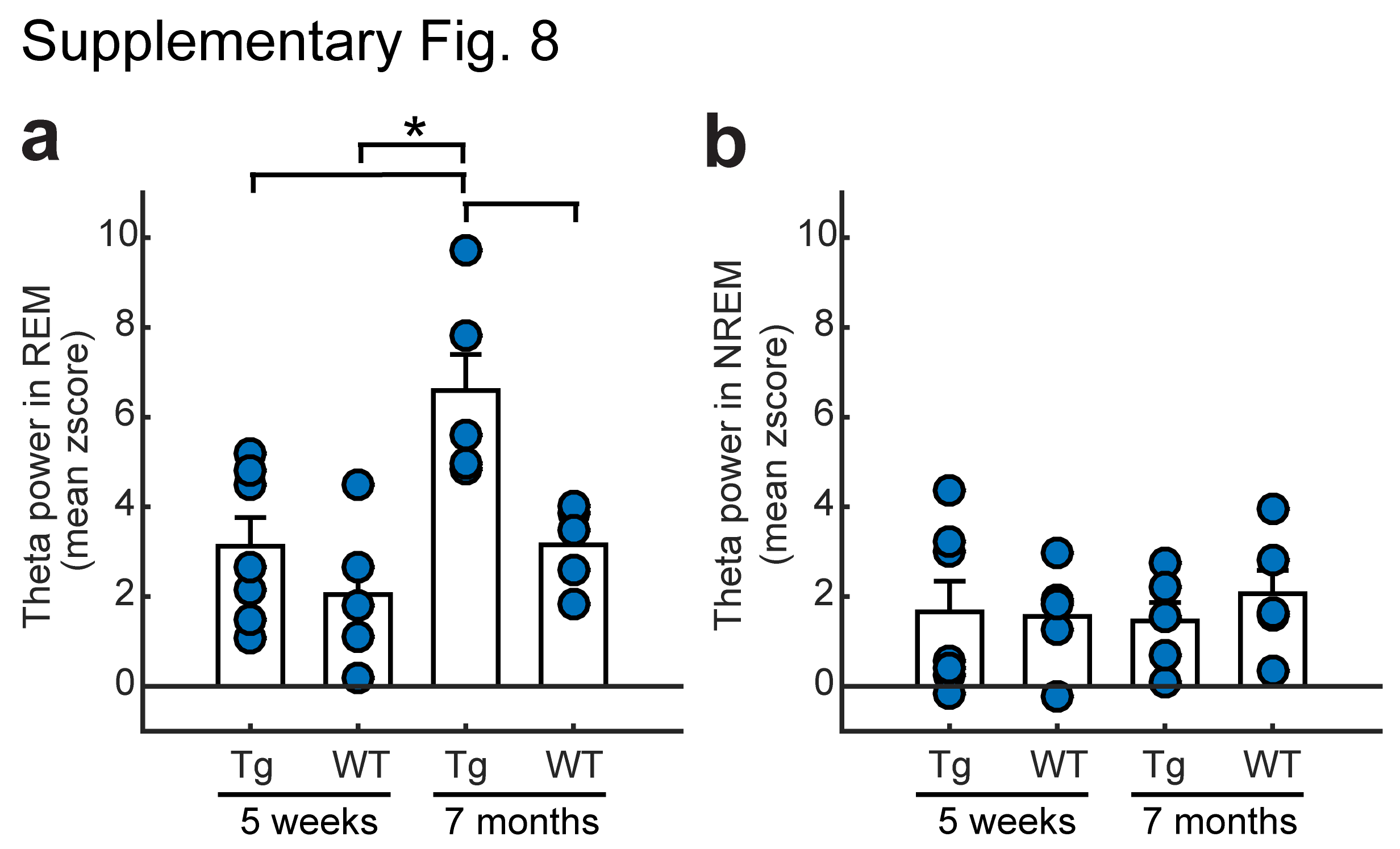


Suppl. Fig. 8 | Theta power in Tg2576 mice is different from WT mice in REM sleep but not different in NREM sleep.

1. Data from Figure 4d are shown for comparative purposes.
2. Quantification of theta power for all NREM sleep epochs in a 24 hour-long recording in 5 week-old WT (n=5) and Tg2576 mice (n=7), and 7 month-old Tg2576 (n=5) and WT mice (n=5). There was no effect of age (two-way ANOVA, F(1,21)0.059, p=0.811) or genotype (F(1,21)0.168, p=0.686).


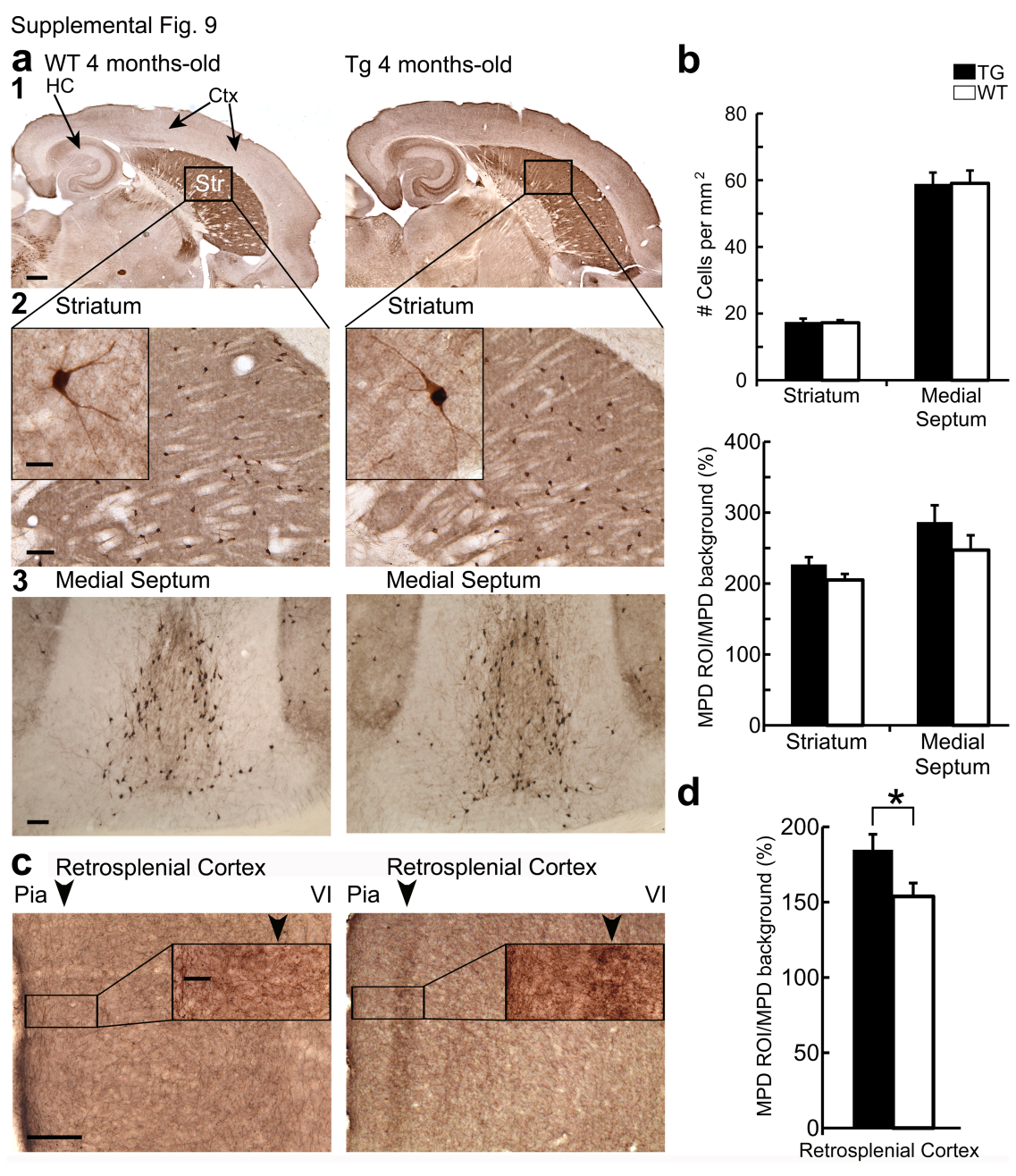


Suppl. Fig. 9 | ChAT-ir in dorsal striatum, medial septum and retrosplenial cortex (RSC).

1. 1. ChAT-ir in a representative 4 month-old WT (left) and Tg2576 mouse (right, TG). Calibration = 500 µm. HC= hippocampus. Str = striatum.

2. The rectangle demarcating an area of the dorsal striatum in **a1** is shown at higher power, for both WT (left) and Tg2576 (right), with an inset displaying representative ChAT labeled striatal neurons. Calibration = 100 µm; insets = 25 µm.

3. ChAT-ir is shown in the medial septum for a WT (left) and Tg2576 mouse (right, TG). The insets show medial septal neurons with ChAT-ir. Calibration = 100 µm.

1. Top: The number of ChAT-ir neurons was calculated per mm^2^ for both the striatum and the medial septum for 4 month-old Tg2576 (black bars; n=15) and WT (white bars, n=11) mice. Two-way ANOVA showed no effect of genotype (F(1,48)0.0003, p=0.986).

Bottom: MPD is shown for 4 month-old Tg2576 and WT mice. MPD is expressed relative to background; MPD of the region of interest (ROI)/ MPD background (%). Two-way ANOVA showed no effect of genotype (F(1,48)2.943, p=0.093).

c) A ChAT-ir labeled area of the retrosplenial cortex is shown for both WT (left) and Tg2576 (right), with an inset at high power displaying the superficial layers (arrowheads). Calibration = 100 µm; insets = 50 µm.

d) The retrosplenial cortex is shown for 4 month-old Tg2576 and WT mice. MPD measurements of the superficial layers showed greater ChAT-ir in the Tg2576 mouse compared to WT (Student’s t-test, p=0.046).


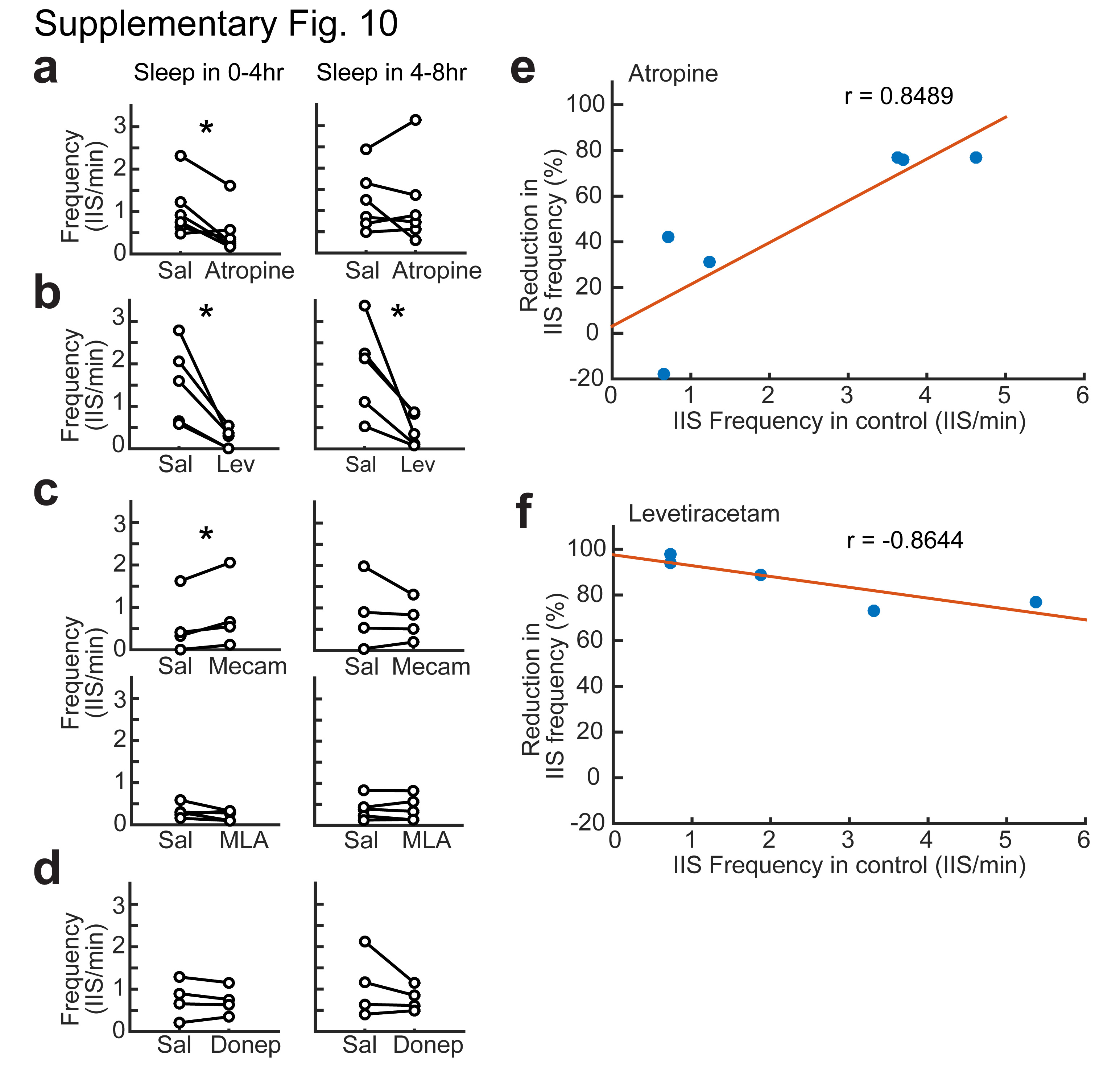


Suppl. Fig. 10 | Pharmacology of IIS in sleep.

1. Left: Atropine significantly decreased IIS frequency during sleep (REM and NREM) in the 0-4 hours after injection compared to the 0-4 hours after saline injection (paired t-test, n=7, p=0.018). Right: IIS frequency 4-8 hours after atropine injection was not significantly different from IIS frequency 4-8 hours after saline injection (paired t-test, n=7, p=0.779).
2. Top: There was a slight but significant increase in IIS frequency in the 0-4 hours following mecamylamine administration (Mecam; paired t-test, n=4, p=0.048). However, there was no longer any difference from control at 4-8 hours after injection (p=0.465). Bottom: There were no significant effects of methylylcaconitine (MLA) 0-4 hours after injection (paired t-test, n=5, p=0.242) or 4-8 hours after injection (p=0.992).
3. There was no effect of donepezil (Donep) on IIS frequency during sleep (paired-t-test, n=4; 0-4 hours: p=0.595; 4-8 hours: p=0.288).
4. Left: Levetiracetam (Lev) significantly decreased IIS frequency during sleep in the 0-4 hours after injection (paired t-test, n=5, p=0.021). Right: IIS frequency in the 4-8 hours after injection of Lev was also reduced (p=0.029), consistent with its long half-life.
5. The magnitude of the effect of atropine (during NREM and REM sleep, pooled, 0-4 hours after atropine treatment) was greater for those mice that had relatively high IIS frequency in REM sleep 0-4 hours after saline injection (Pearson's correlation coefficient r=0.849, p=0.032). X-axis: IIS frequency during REM, 0-4 hours after saline injection. Y-axis: percent reduction in IIS frequency by atropine compared to control [(IIS frequency in NREM and REM sleep, 0-4 hours after saline injection) - (IIS frequency in NREM and REM sleep 0-4 hours after atropine)] / (IIS frequency in NREM and REM sleep, 0-4 hours after saline).
6. The correlation for levetiracetam was not significant (Pearson's correlation coefficient r=-0.864, p=0.059) using same calculation as in **e**.


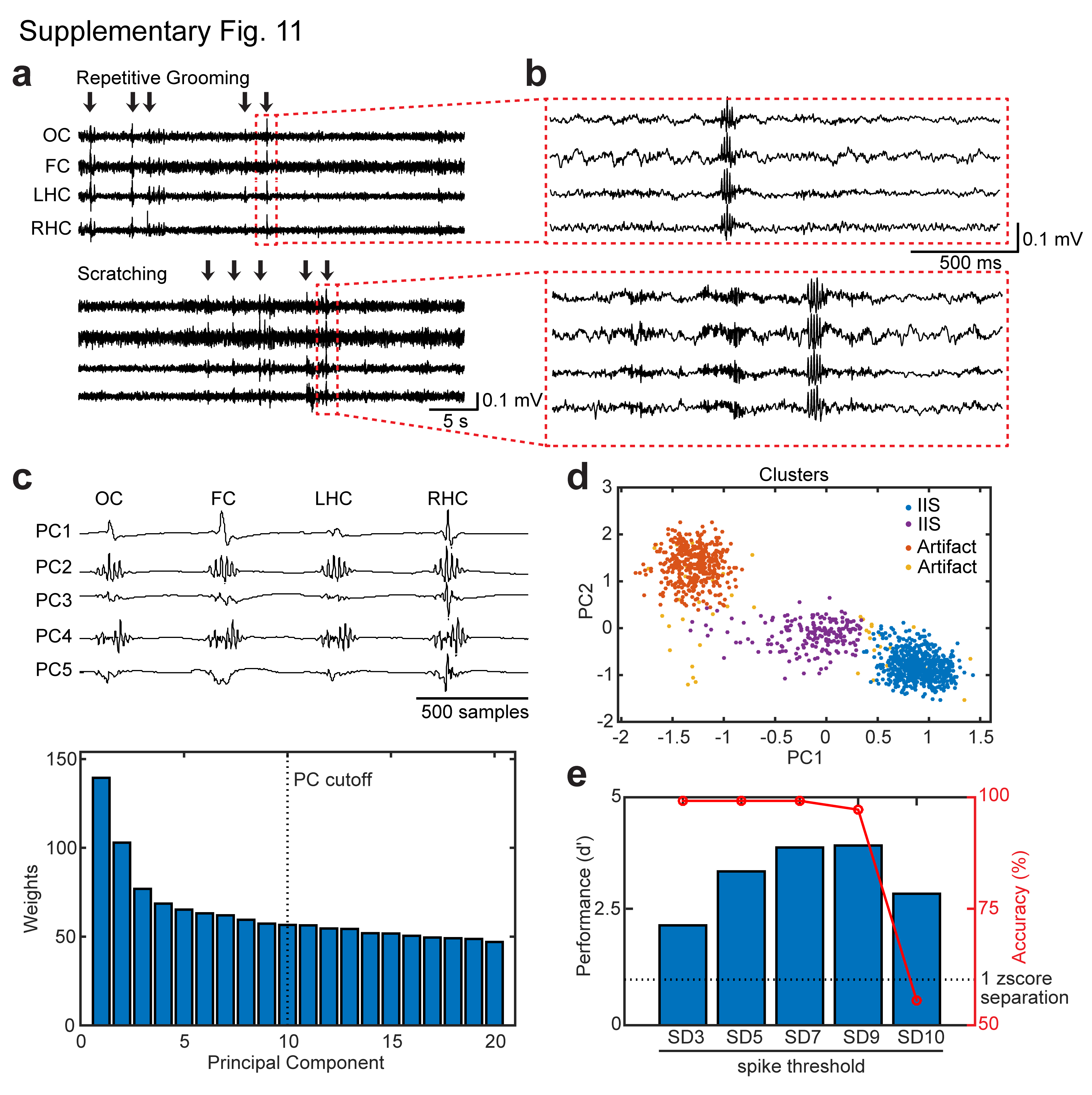


Suppl. Fig. 11 | IIS detection in Tg2576 mice.

1. Examples of artifacts in EEG recordings. Artifacts typically occurred during repetitive grooming or scratching of the head.
2. The area outlined by the dotted red lines in **a** is expanded.
3. Top: An example of the first five principal components (PC) in a 24 hour recording. Bottom: PCs are plotted with associated weights (relative amplitude contribution). The first ten PCs were sufficient to distinguish IIS from artifacts, indicated by the dotted line.
4. A scatter plot shows k-means clustering to discriminate IIS (blue, purple) from artifacts (orange, red).
5. The performance of automated IIS detection was compared to manual identification in two ways. Left Y axis: performance (d’; blue bars left Y axis), a signal detection theory metric, was used, where higher bars indicate greater detection sensitivity. Each blue bar designates the performance of a different set of IIS templates for automated detection compared to manual review, with the IIS detection threshold set at 3-10 standard deviations (SD) greater than the amplitude of baseline noise. Based on this evaluation, 7 zscores was chosen. Right Y axis (red): the accuracy (percent of IIS correctly detected relative to all IIS determined by manual review) is plotted.
